# Aging alters the effect of adiponectin receptor signaling on bone marrow‐derived mesenchymal stem cells

**DOI:** 10.1111/acel.14390

**Published:** 2024-10-27

**Authors:** Hanghang Liu, Qiucheng Zhao, Shibo Liu, Bolun Li, Zizhuo Zheng, Yao Liu, Pei Hu, En Luo

**Affiliations:** ^1^ State Key Laboratory of Oral Diseases & National Center for Stomatology & National Clinical Research Center for Oral Diseases, West China Hospital of Stomatology Sichuan University Chengdu Sichuan China; ^2^ MaineHealth Institute for Research Scarborough Maine USA; ^3^ Suzhou Stomatological Hospital Suzhou Jiangsu People's Republic of China

**Keywords:** aging, fracture prevention, molecular pathways–remodeling, osteoporosis, stromal/stem cells

## Abstract

Adiponectin receptor signaling represents a promising therapeutic target for age‐related conditions such as osteoporosis and diabetes. However, the literature presents conflicting evidence regarding the role of adiponectin signaling in bone homeostasis and fracture repair across different health states, ages, and disease conditions. These inconsistencies may arise from the complex endocrine and paracrine feedback mechanisms regulating adiponectin, as well as the variability in adiponectin isoforms and receptor expressions. In this study, we observed differential expression of adiponectin receptors in the bone marrow (BM) of aged mice, characterized by elevated levels of adiponectin receptor 2 and reduced levels of receptor 1, as corroborated by both single‐cell sequencing and in vivo staining. Additionally, circulating levels of adiponectin and its local expression were significantly higher in aged mice compared to younger counterparts. Treatment with adiponectin receptor agonist, AdipoRon, enhanced bone regeneration and repair in young mice by promoting osteogenesis and reducing osteoclastogenesis. Conversely, in aged mice, AdipoRon treatment led to cellular senescence, delayed bone repair, and inhibited osteogenic activity. Notably, the adiponectin receptor 1‐Wnt and adiponectin receptor 2‐MAPK and mTOR signaling pathways were differentially activated in AdipoRon‐treated BM mesenchymal stem cells from young and aged mice. Additionally, the NF‐κB, and AKT pathways were consistently downregulated in BM macrophages of both age groups following AdipoRon administration. In conclusion, aging significantly modulates the impact of adiponectin receptor signaling on BM mesenchymal stem cells. This modulation is potentially attributable to changes in receptor transcription and distribution, as well as differential activation of downstream signaling pathways.

## Graphical abstract

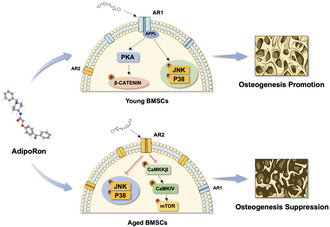

**Abbreviations**
ALP: alkaline phosphatase
AMPK: AMP‐activated protein kinase
APN: adiponectin
APR: AdipoRon
AR1: adiponectin receptor 1
AR2: adiponectin receptor 2
ARS: alizarin red staining
Atc‐D: actinomycin D
BFR: bone formation rate
BM: bone marrow
BMA: bone marrow adipocyte
BMD: bone mineral density
BMMs: bone marrow‐derived macrophages
BMSCs: bone marrow‐derived mesenchymal stem cells
BV/TV: bone volume/total volume ratio
CCCs: cell–cell communications
CHX: cycloheximide
Ct.Th.: cortical bone thickness
DEGs: differentially expressed genes
FBS: fetal bovine serum
IGV: Integrative genomics viewer
MAPK: Mitogen‐activated protein kinase
MAR: mineral apposition rate
MS/BS: mineral surface/bone surface
MSC: mesenchymal stem cell
OB: osteoblasts
OC: osteoclast
OVX: ovariectomy
qPCR: quantitative polymerase chain reaction
ROI: regions of interest
ROS: reactive oxygen species
scRNA‐seq: single‐cell RNA sequencing
Tb.N: trabecular number
Tb.Sp: trabecular separation
t‐SNE: T‐distributed stochastic neighbor embedding

## 1 INTRODUCTION

Aging is a multifaceted process characterized by the progressive loss of structural integrity in various organs and tissues. This deterioration is frequently accompanied by chronic inflammation and a heightened risk of age‐associated diseases, including osteoporosis, sarcopenia, and cancer (Abdelmagid et al., 2015). During aging, bone marrow (BM) undergoes a gradual replacement by adipose tissue. This substitution contributes to an increase in local adipocyte‐derived cytokines, coupled with reductions in bone mass and strength (Takeshita et al., 2014). Consequently, in humans, diminished bone mass is linked to an elevated susceptibility to microfractures (Ensrud, 2013).

A previous clinical study indicated that both age and sex independently correlate with serum adiponectin (APN) levels (Tomono et al., 2018). Analyses reveal a positive association between APN levels and both age and cholesterol levels (Bik et al., 2013; Meshkini et al., 2018; Obata et al., 2013). Centenarians exhibit significantly higher circulating APN and lower leptin levels compared to younger individuals (Meazza et al., 2011). Serum APN concentrations are lower in men than in women (Meshkini et al., 2018; Obata et al., 2013), and testosterone treatments have been shown to decrease circulating APN levels (Duque, 2008). Additionally, it was suggested that elevated APN levels are inversely associated with bone strength and maximal load capacity in middle‐aged men (Tan et al., 2014). Given that BM increasingly accumulates adipocytes during aging, and that APN secretion from BM adipocytes (BMA) surpasses that from white adipose tissue (Cawthorn et al., 2014), it is plausible that the age‐related increase in BMA secreted APN significantly impacts the functionality of BM‐derived mesenchymal stem cells (BMSCs). Proposed explanations for the age‐associated rise in APN levels include diminished sex hormone levels and reduced efficacy of APN receptors (Obata et al., 2013). Although elevated serum APN levels in the elderly or postmenopausal women may not yield numerous beneficial effects (Lewis et al., 2021), the specific mechanisms regulating the differential impacts of APN across age groups remain elusive and warrant further exploration.

APN receptors 1 and 2 (AR1 and AR2) are the primary mediators of APN's biological functions. These receptors are expressed across the mesenchymal stem cell (MSC) lineage, including BMSCs, osteoblasts (OB), and chondrocytes. Although T‐cadherin, a newly identified APN receptor, plays a pivotal role in vascular endothelial cells, hamster ovary cells, and C2C12 myoblasts (Fukuda et al., 2017; Kita et al., 2019), AR1 and AR2 knockout could almost wholly block the actions of APN in mice (Yamauchi et al., 2007). In the past decades, the impact of APN on bone homeostasis has been thoroughly investigated through both in vivo and in vitro studies in humans and mice, focusing on potential therapeutic targets in disease states. While several in vitro studies suggest that APN can stimulate OB differentiation (Chen et al., 2015; Huang et al., 2010; Lee et al., 2009), impair preadipocyte differentiation (Yan et al., 2013), reduce lipid accumulation (Lazra et al., 2015), and inhibit osteoclast (OC) formation (Ebina et al., 2009; Tu et al., 2011; Yamaguchi et al., 2007), numerous animal and clinical studies have demonstrated a negative correlation between serum APN and whole‐body mineral content (Bozic et al., 2010; Peng et al., 2008). Intriguingly, higher circulating APN levels have been associated with reduced bone mass acquisition, and mice with a genetic deletion of APN exhibit increased bone mass (Williams et al., 2009; Yang et al., 2019). The absence of APN or consistently low levels of full‐length APN also appear to protect against bone loss induced by ovariectomy (OVX) in the mandible, suggesting a protective role against menopause‐induced bone resorption (Lewis et al., 2021). Despite this wealth of data, the existing research has predominantly focused on juvenile or adult models; the effects of APN and AR signaling in aging or osteoporotic bone development related to aging remain unexplored. Critical questions persist: What is the impact of APN receptor signaling on osteogenic bone formation, and how does AR signaling influence bone repair as age advances.

AdipoRon (APR) is an orally active small molecule that acts as an agonist for both AR1 and AR2, which was firstly identified by Okada‐Iwabu et al. (2013). The discovery of APR has facilitated detailed investigations into the AR signaling pathway and its influence on bone formation, independent of the endocrine/paracrine feedback mechanisms and variable APN protein‐receptor interactions, as previously noted. In the most recent studies, APR exhibits effects akin to those of APN, such as reducing ischemia/reperfusion‐induced cardiac injury and cardiomyocyte cell death (Botta et al., 2019; Fairaq et al., 2017), suppressing tumor growth (Akimoto et al., 2018; Ramzan et al., 2019; Sapio et al., 2020), and promoting repair in brain tissue and neural cells (Liu et al., 2020; Ng et al., 2020; Yu et al., 2019). Additionally, APR treatment has been shown to enhance the survival and migration of BMSCs (Malih et al., 2017; Wu et al., 2019), support the survival and differentiation of chondrocytes (Wang et al., 2019), inhibit adipogenesis (Wang et al., 2017) and improve skeletal muscle function (Balasubramanian et al., 2022; Botta et al., 2020; Ito et al., 2018).

In our research, in order to exploring how aging influences APN signaling in bone remodeling, we investigated the expression level of APN and its receptors in young and aged mice via both single cell sequencing and in vivo staining. We also examined the effects of APR on bone regeneration and OC formation in both young and aged mice groups through in vitro and in vivo methods, aiming to understand the potential role of AR signaling in aging‐related osteoporosis. This study provides mechanistic insights into the beneficial and pathological effects of AR signaling and evaluates the therapeutic potential of targeting APN or AR signaling in treating aging‐related bone abnormalities and promoting bone repair.

## 2 RESULTS

### 2.1 Aged mice showed lower bone density and higher serum circulating APN

**FIGURE 1:**
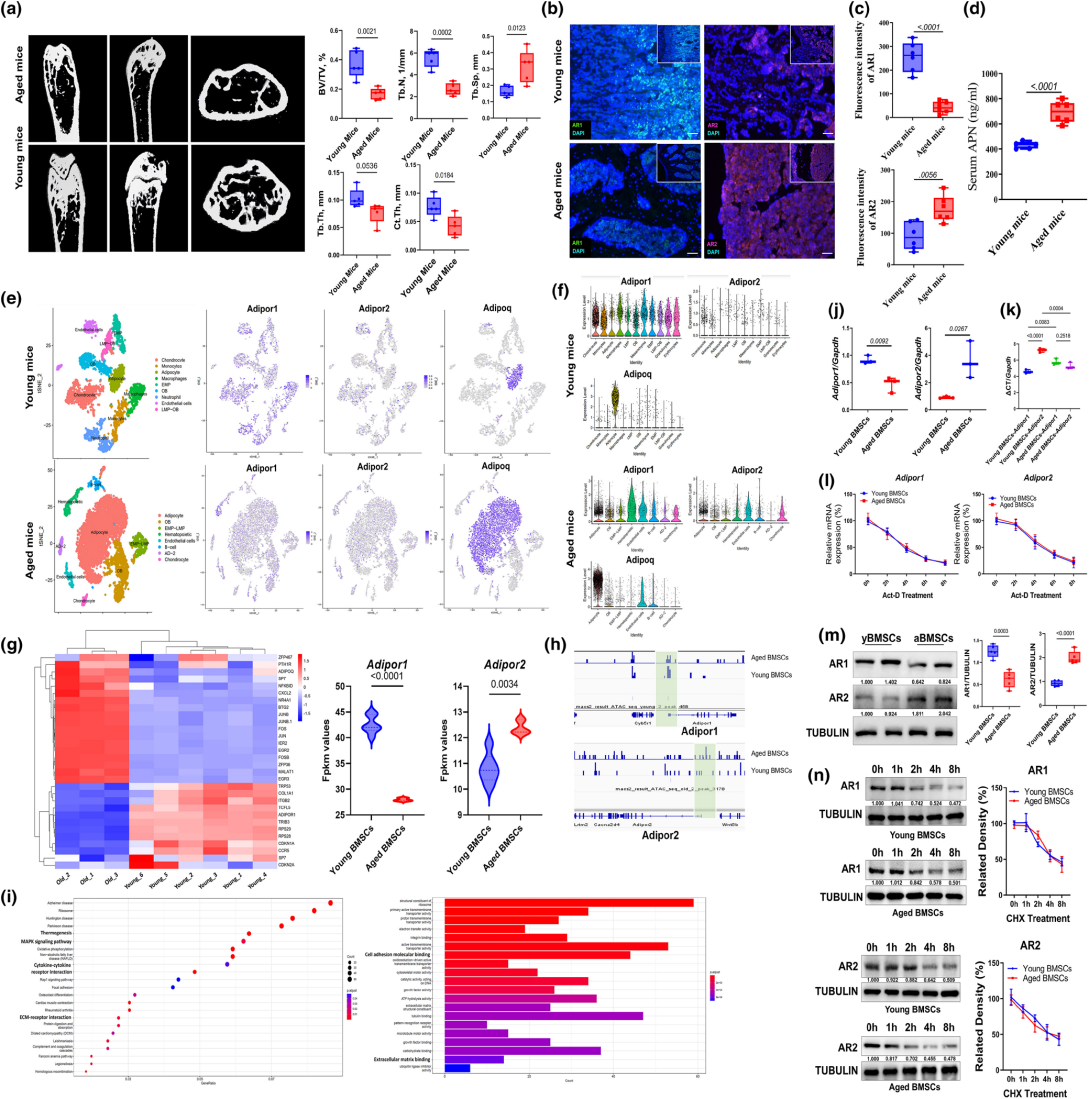
Serum APN level and ARs expression level in young and aged mice. (a) Representative image of micro‐CT and related quantitative analysis of femur in 3‐ and 20‐month‐old male mice. Data shown as mean ± SD, *n* = 6 per group; (b and c) Immunofluorescence staining and related quantitative results of AR1 and AR2 in femur bone marrow of 3‐ and 20‐month‐old male mice. Data shown as mean ± SD, *n* = 6 per group; scale bar, 50 μm; (d) Elisa results of serum APN level in 3‐ and 20‐month‐old male mice. Data shown as mean ± SD, *n* = 6 per group; (e) t‐SNE plot of scRNA‐seq showing the cell clusters in Col2+ mesenchymal lineage cells isolated from endosteal bone marrow of 3‐month‐old (above) and 16‐month old (bottom) mice, and the expression of *Adipor1*, *Adipor2*, and *Adiponectin* in different clusters. (f) Violin plots of *Adipor1*, *Adipor2*, and *Adiponectin* gene expression in different clusters in in Col2+ mesenchymal lineage cells isolated from endosteal bone marrow of 3‐month old (above) and 16‐month old (bottom) mice. (g) Heat map of differentially expressed genes (DEG) and expression level expressed as RPKM values of *Adipor1* and *Adipor2* detected by RNA‐seq between BMSCs isolated from 3‐ and 16‐month old mice. (h) Integrative genomics viewer (IGV) browser views showing ATAC‐seq in young and aged BMSCs, near the *Adipor1* and *Adipor2* genes. (i) Representation of GO and KEGG terms analyzed with overlapping genes identified by RNA‐seq and ATAC‐seq. (j) qPCR results of *Adipor1* and *Adipor2* in BMSC derived from 6‐week‐old and 20‐month‐old male mice, *n* = 3 independent experiments. (k) Relative ΔCT value of *Adipor1* and *Adipor2* in BMSC derived from 6‐week‐old and 20‐month‐old male mice. Data shown as mean ± SD, *n* = 3 independent experiments. (l) qPCR results of *Adipor1* and *Adipor2* in BMSC derived from 6‐week‐old and 20‐month‐old male mice with 0, 2, 4, 6, and 8 h 10 μg/mL Act‐D treatment. Data shown as mean ± SD, *n* = 3 independent experiments. (m) Immunoblot and related quantitative results of AR1 and AR2 in BMSC derived from 6‐week‐old and 20‐month‐old male mice. Data shown as mean ± SD, *n* = 3 independent experiments. (n) Immunoblot results and related turnover rate of of AR1 and AR2 in BMSC derived from 6‐week‐old and 20‐month‐old male mice with 0, 1, 2, 4, and 8 h 50 μg/mL CHX treatment. Data shown as mean ± SD, *n* = 3 independent experiments. yBMSCs, young BMSC; aBMSC, aged BMSCs. APN, adiponectin.

To investigate age‐related differences in serum APN levels and AR expression in BM of mice, we utilized ELISA, immunofluorescence staining, and western blotting methods. As expected, 20‐month‐old mice exhibited osteoporosis with significantly lower femur bone volume ratio (BV/TV) (*p* = 0.0021), trabecular number (Tb. N) (*p* = 0.0002), cortical bone thickness (Ct. Th) (*p* = 0.0184), alongside higher trabecular separation (Tb. Sp) (*p* = 0.0123) compared to 3‐month‐old control (Figure 1a). Interestingly, immunofluorescence staining revealed dramatically higher AR1 expression (*p* < 0.0001) and substantially lower AR2 expression (*p* = 0.0056) in the BM of young mice compared to aged mice (Figure 1b,c). Consistently, our results confirmed significantly elevated serum APN levels in aged mice compared to young mice (Figure 1d).

In order to confirm these changes, we re‐analyzed previous single‐cell RNA sequencing (scRNA‐seq) from GSE145477 datasets (Figure S1). In Zhong's study (2020), they conducted single‐cell RNA‐seq derived from Td + endosteal BM cells of 3‐ and 16‐month‐old Col2 mice. Col2 was employed as a novel marker to identify specific mesenchymal subpopulations within the BM. These cells were collected via enzymatic digestion, followed by single‐cell sequencing using the 10× Genomics platform. Through this dataset, we are able to identified potential differences in BMSCs between young and aged mice, specifically focusing on the expression levels of adiponectin signaling pathways and the distribution of adiponectin receptors. These findings may support our hypothesis that aging may influence adiponectin receptor distribution and signaling within the BM. Nor surprisingly, the results revealed significant differential expression of key regulators in the adiponectin signaling pathway in Col2+ mesenchymal lineage cells that isolated from the endosteal BM of 3‐ versus 16‐month‐old mice (Figure 1e). Subsequent analyses, illustrated with violin plots, showed a decrease in *Adipor1* and an increase in *Adipor2* expression in the 16‐month‐old mice compared to the 3‐month‐old cohort, particularly in early and late mesenchymal progenitors and OB. Notably, *Adipoq* was primarily expressed in differentiated adipocytes, with a significant trend toward increased expression in the 16‐month‐old mice relative to the younger control (Figure 1f). These findings align with previous clinical studies and provide valuable insights into the differential regulation of APN and its receptors with aging.

Additionally, we also re‐analyzed previous bulk RNA‐seq data from the GSE143580 dataset. In the study by Andromachi Pouikli et al., (2021), BMSCs were isolated from the endosteum of 3‐ and 20‐month‐old mice and subsequently purified through passaging. The expression profiles of both young and aged BMSCs were analyzed using RNA‐seq, while global chromatin accessibility was assessed via ATAC‐seq. By re‐analyzing this dataset, we could confirm the differential transcriptional patterns of adiponectin receptors between young and aged BMSCs, further supporting our hypothesis that aging may affect adiponectin receptor distribution and signaling within the BM. Interestingly, the results revealed a significantly reduced transcriptional level of *Adipor1* and an increased transcriptional level of *Adipor2* in aged BMSCs compared to young BMSCs (Figure 1g). To investigate whether specific genomic loci of *Adipor1* and *Adipor2* exhibited altered chromatin accessibility with aging, we conducted a combined analysis with ATAC‐seq data. Our observations indicated that the gene promoters in aged cells showed substantial differences in chromatin accessibility, which is closely linked to transcriptional output. As expected, a significant loss of chromatin accessibility at the *Adipor1* promoter was noted in aged BMSCs, whereas a much higher promoter accessibility was observed for *Adipor2* (Figure 1h). KEGG and GO enrichment analysis of genes associated with promoter and enhancer regions between young and aged BMSCs highlighted that aged BMSCs displayed reduced promoter accessibility for terms related to mesenchymal cell differentiation, MAPK signaling, and cell‐matrix adhesion processes, consistent with the transcriptional changes observed in the RNA‐seq data (Figure 1i).

To validate this finding, we further isolated BMSCs from both young and aged mice to assess the expression levels of AR1 and AR2 using qPCR and Western blotting. The qPCR analysis revealed that aged BMSCs exhibited higher transcriptional levels of *Adipor2* but lower levels of *Adipor1* compared to young BMSCs (Figure 1j). Notably, the relative ΔCT value for AdipoR1 was slightly lower in young BMSCs than in aged BMSCs, whereas for AdipoR2, it was higher (Figure 1k). These findings suggest that AR1 predominantly mediates the effects of APN in young cells, while AR2 plays a larger role in aging cells. To examine RNA stability, we treated young and aged BMSCs with actinomycin D and observed no significant differences in the stability of *Adipor1* and *Adipor2* transcripts over time intervals of 0, 2, 4, 6, and 8 h (Figure 1l). Additionally, protein analysis showed higher levels of AR2 and lower levels of AR1 in BMSCs from aged mice compared to those from young mice (Figure 1m). Upon inhibiting protein synthesis with cycloheximide (CHX), we determined that the turnover rates of AR1 and AR2 proteins were consistent between young and aged BMSCs (Figure 1n). Combined with ATAC‐seq data, these results implied that the differences in AR1 and AR2 expression between young and aged BMSCs are attributable to differential transcriptional activities, potentially influenced by epigenetic modifications, such as DNA methylation and histone changes in aged cells (Jae‐Hyun Yang et al., 2023). Furthermore, AR1 expression was similar between young and aged BM‐derived macrophages (BMMs) from mice, yet notably lower than that observed in BMSCs. Conversely, AR2 levels were significantly reduced in BMMs derived from aged mice compared to those from young mice (Figure S2).

### 2.2 APR promoted the repair of skull and femur defects in young mice

**FIGURE 2:**
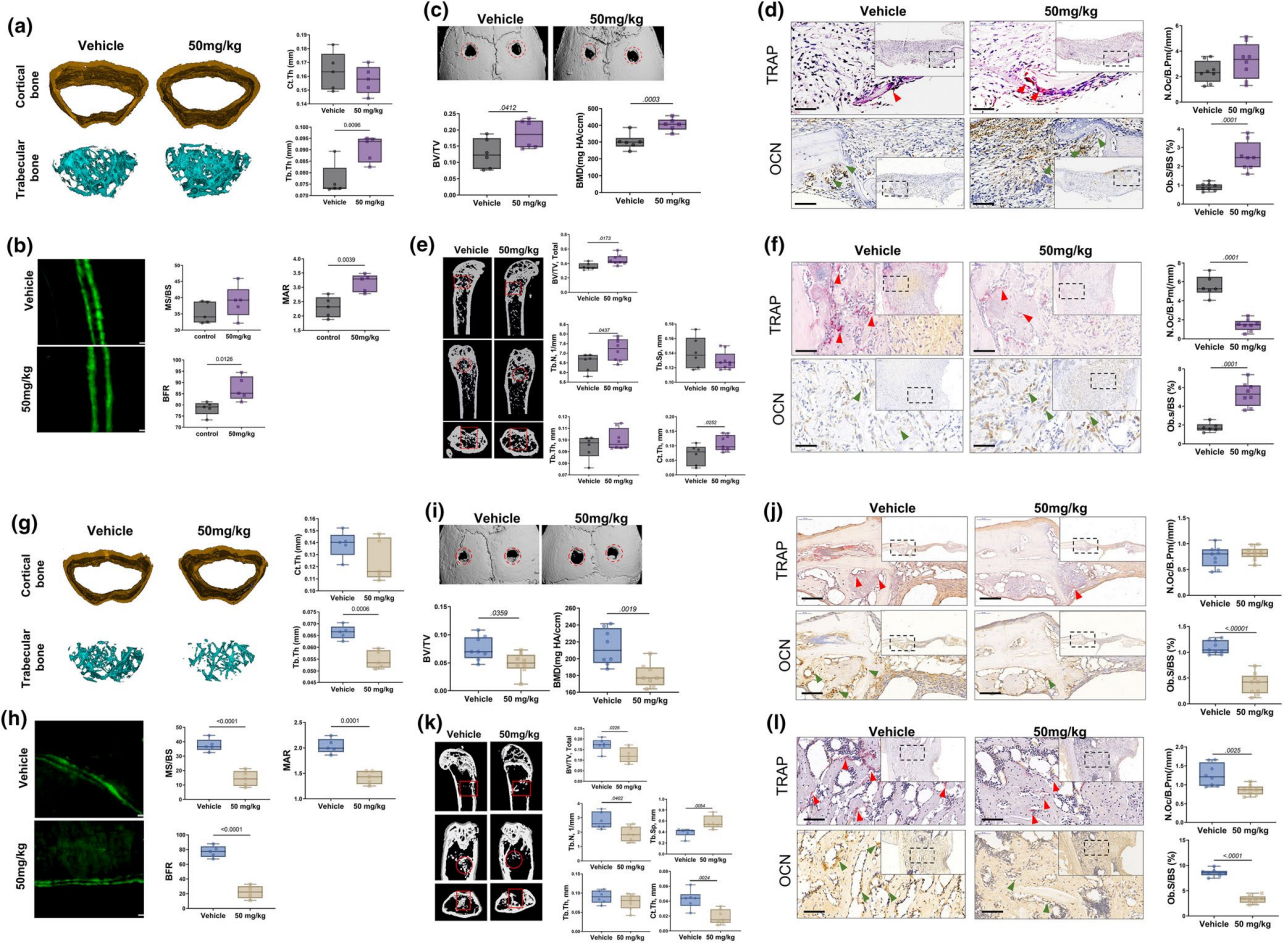
APR promoted skull and femur defect repairing in young and aged male mice. (a) Representative images of micro‐CT and related quantitative analysis of femur cortical and trabecular bone in young mice at 2‐week after APR treatment. Data shown as mean ± SD, *n* = 5 per group. (b) Dynamic histomorphometry images and related quantitative analysis of femur trabecular and cortical bones in young mice at 2‐week after APR treatment. Data shown as mean ± SD, *n* = 5 per group. Scale bar: 10 μm. (c) Representative images of micro‐CT and related quantitative analysis of skull at 2‐week after surgery, red cycle: Bone defect region; Data shown as mean ± SD, *n* = 5–8 per group. (d) Representative TRAP and OCN‐immunochemistry staining pictures and related quantitative analysis of skull defect at 2‐week after surgery. Red arrow: osteoclast, green arrow: osteoblast. Scale bar: 50 μm; (e) Representative images of micro‐CT and related quantitative analysis of femur at 2‐week after surgery, red cycle/rectangular: Bone defect region; Data shown as mean ± SD, *n* = 5–8 per group. (f) Representative TRAP and OCN‐immunochemistry staining pictures and related quantitative analysis of femur defect at 2‐week after surgery. Red arrow: osteoclast, green arrow: osteoblast; Data shown as mean ± SD, *n* = 5–8 per group. Scale bar: 50 μm. (g) Representative images of micro‐CT and related quantitative analysis of femur cortical and trabecular bone in aged mice at 2‐week after APR treatment. Data shown as mean ± SD, *n* = 5 per group. (h) Dynamic histomorphometry images and related quantitative analysis of femur trabecular and cortical bones in young mice at 2‐week after APR treatment. Data shown as mean ± SD, *n* = 5 per group. Scale bar: 10 μm. (i) Representative images of micro‐CT and related quantitative analysis of skull at 2‐week after surgery, red cycle: Bone defect region; Data shown as mean ± SD, *n* = 5–8 per group. (j) Representative TRAP and OCN‐immunochemistry staining pictures and related quantitative analysis of skull defect at 2‐week after surgery. Red arrow: osteoclast, green arrow: osteoblast; Data shown as mean ± SD, *n* = 5–8 per group. Scale bar: 50 μm. (k) Representative images of micro‐CT and related quantitative analysis of femur at 2‐week after surgery, red cycle/rectangular: bone defect region; Data shown as mean ± SD, *n* = 5–8 per group. (l) Representative TRAP and OCN‐immunochemistry staining pictures and related quantitative analysis of femur defect at 2‐week after surgery. Red arrow: osteoclast, green arrow: osteoblast. Data shown as mean ± SD, *n* = 5–8 per group. Scale bar: 50 μm. BFR/BS, bone formation rate; BV/TV, bone volume/total volume; Ct.Th, cortical bone thickness; MAR, mineral apposition rate; MS/BS, mineral surface/bone surface; N.Oc/B.Pm, number of osteoclast/bone perimeter; Ob.S/BS, osteoblast surface/bone surface; Tb.N, trabecular number; Tb.Sp., trabecular separation; Tb.Th, Trabecular bone thickness.

To investigate the differential effects of AR activation on bone regeneration and defect repair between young and aged mice, we subjected both age groups to standard care or bone defect surgery, followed by treatment with or without APR daily for 2 weeks. In the normal group of 3‐month‐old mice, micro‐CT analysis was conducted on the femoral metaphysis following 2 weeks of APR treatment. Although cortical bone thickness did not differ significantly between groups, trabecular bone thickness was significantly higher in the APR‐treated group compared to the vehicle control (*p* = 0.0096) (Figure 2a). To elucidate the cellular mechanisms underlying the increased bone mass observed in APR‐treated young mice, dynamic histomorphometry were performed on the proximal femur. These analyses revealed slightly elevated mineral surface/bone surface (MS/BS), and significantly increased mineral apposition rate (MAR, *p* = 0.0039), and bone formation rate (BFR, *p* = 0.0126) in the APR group (Figure 2b). Consistently, in the bone defect model, micro‐CT analysis revealed that the APR group exhibited significantly higher total BV/TV (*p* = 0.0412) and bone mineral density (BMD) (*p* = 0.0003) than the vehicle group after the 2‐week treatment in the skull defect model (Figure 2c). Immunohistochemistry results revealed that the APR groups exhibited a significant increase in the number of OCN‐positive cells and a higher osteoblast surface/bone surface ratio (*p* = 0.0001) compared to the vehicle group. Conversely, TRAP activity, a marker of OC activity, showed no significant difference in the skull defect at 2 weeks post‐treatment (Figure 2d).

Similar observations were noted in the femur defect model during the repair process. micro‐CT analysis conducted 2 weeks post‐surgery showed that the APR‐treated group exhibited a significant increase in total BV/TV (*p* = 0.0173), Tb. N (*p* = 0.0437), and Ct. Th (*p* = 0.0252), compared to the vehicle group. However, Tb. Th and Tb. Sp did not show significant differences (Figure 2e). Immunohistochemical analyses further supported these findings, indicating a decrease in the number of TRAP‐positive cells (*p* = 0.0001) and an increase in the osteoblast surface/bone surface ratio (*p* = 0.0001) in the APR group after 2 weeks of treatment (Figure 2f).

### 2.3 APR inhibited skull and femur defect repair in aged mice

Similar experiments were conducted with 20‐month‐old male mice. In those without bone defects, micro‐CT analysis demonstrated a significant reduction in trabecular bone thickness in aged mice treated with APR compared to those receiving the vehicle (*p* = 0.0006); however, cortical bone thickness did not show substantial differences (Figure 2g **).** Additionally, dynamic histomorphometry data confirmed that APR treatment significantly decreased MS/BS (*p* < 0.0001), MAR (*p* = 0.0001), and BFR (*p* < 0.0001) (Figure 2h). Following bone defect surgery, daily gavage of mice with or without APR for 2 weeks demonstrated that the APR group exhibited significantly lower total BV/TV (*p =* 0.0026) and BMD (*p =* 0.0019) compared to the vehicle group in the skull defect model (Figure 2i). Notably, no obvious active OCs were observed in the bone defect, and there was no difference in TRAP‐positive cells between the two groups (Figure 2j). However, a markedly lower OCN‐positive cell count and osteoblast surface/bone surface ratio were noted at 2 weeks post‐APR treatment (*p <* 0.0001) (Figure 2j).

Further analysis using micro‐CT in the femur defect repair model revealed a slight decrease in total BV/TV (*p =* 0.0226), decreased Tb. N (*p =* 0.0402), Ct. Th (*p =* 0.0024), and an significantly increase in Tb. Sp (*p =* 0.0054) in the APR group compared to the vehicle group at 2 weeks after surgery. However, no differences were observed in Tb. Th between the two groups (Figure 2k). Importantly, significantly lower numbers of OCs (*p =* 0.0025) as well as a reduced osteoblast surface/bone surface ratio (*p <* 0.0001) were observed after 2 weeks of APR treatment in the femur (Figure 2l), indicating diminished bone remodeling in the APR group.

### 2.4 APR promoted cell migration and osteogenesis of young mouse‐derived BMSCs but showed the opposite effect on aged mouse‐derived BMSCs

**FIGURE 3:**
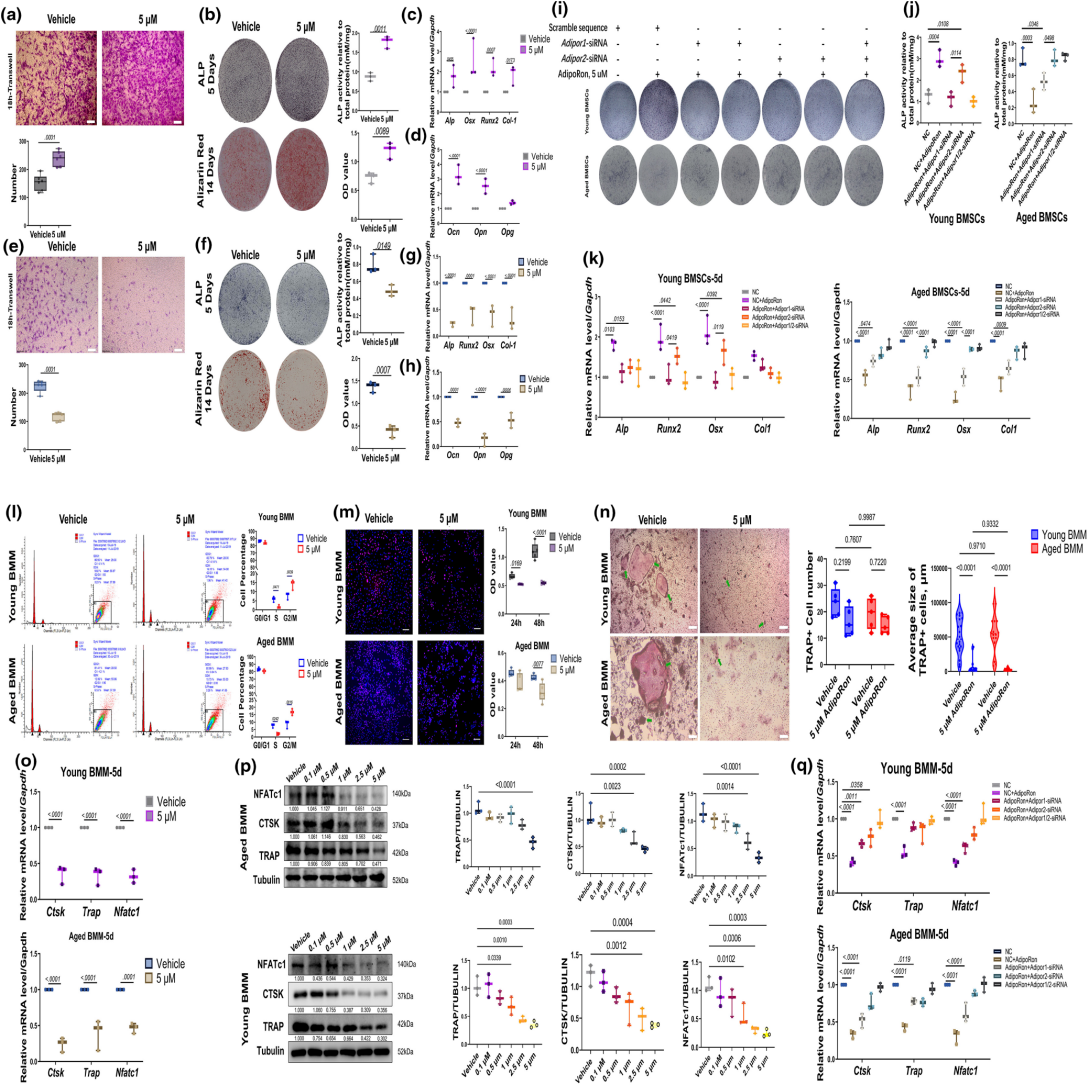
Effect of AdipoRon on young and aged BMSCs and BMM. (a) Representative image of transwell assay for young BMSCs after 18 h APR treatment and related quantitative analysis. Data shown as mean ± SD, *n* = 3 independent experiments; Scale bar: 200 μm. (b) Representative images of ALP staining of young BMSCs after 5 days' OB differentiation, and images of AR staining of young BMSCs after 14 days' OB differentiation, and related quantitative. Data shown as mean ± SD, *n* = 3 independent experiments; (c) qPCR results of young BMSCs after 5 days' OB differentiation. Data shown as mean ± SD, *n* = 3 independent experiments; (d) qPCR of young BMSCs after 14 days' OB differentiation, *n* = 3 independent experiments; (e) Representative images of transwell assay for aged BMSCs after 18 h APR treatment and related quantitative analysis. Data shown as mean ± SD, *n* = 5 independent experiments. Scale bar: 200 μm. (f) Representative images of ALP staining of aged BMSCs after 5 days' OB differentiation, and images of AR staining of aged BMSCs after 14 days' OB differentiation, and related quantitative. Data shown as mean ± SD, *n* = 3 independent experiments; (g) qPCR results of aged BMSCs after 5 days' OB differentiation. Data shown as mean ± SD, *n* = 3 independent experiments. (h) qPCR of aged BMSCs after 14 days' OB differentiation. Data shown as mean ± SD, *n* = 3 independent experiments; (i and j) Representative images of ALP staining of young and aged BMSCs after 5 days' OB differentiation and APR treatment followed by 24 h siRNA treatment, and related ALP activity quantitative. Data shown as mean ± SD, *n* = 3 independent experiments; (k) qPCR results of osteogenseis related genes for young and aged BMSCs after 5 days' OB differentiation and APR treatment followed by 24 h siRNA treatment. Data shown as mean ± SD, *n* = 3 independent experiments; (l) Cell cycle/apoptosis flow cytometry analysis of young and aged BMM after 24 h APR treatment and related quantitative analysis. Data shown as mean ± SD, *n* = 3 independent experiments; (m) Representative pictures for EdU staining of young and aged BMM after 24 h APR treatment and 24 h EdU incubation (with APR), white scale bar: 250 um. Data shown as mean ± SD, *n* = 5 independent experiments; CCK‐8 assay of young and aged BMSCs after 24‐ and 48‐h APR treatment. Data shown as mean ± SD, *n* = 5 independent experiments; (n) Representative images and quantitative results of TRAP staining of young and aged BMM after 7 days' OC differentiation. Green arrow: osteoclasts. Data shown as mean ± SD, *n* = 5 independent experiments; Scale bar: 200 μm. (o) qPCR results of young and aged BMM after 5 days' OC differentiation. Data shown as mean ± SD, *n* = 3 independent experiments; (p) western blot and related quantitative results of young and aged BMM after 5 days' OC differentiation. Data shown as mean ± SD, *n* = 3 independent experiments; (q) qPCR results of osteoclatogenesis related genes for young and aged BMM after 5 days' OC differentiation followed by 24 h siRNA treatment. Data shown as mean ± SD, *n* = 3 independent experiments.

To determine the factors contributing to the differential response to AR activation between young and aged mice, we conducted in vitro studies comparing cell viability, differentiation, and signaling pathways activation in BMMs and BMSCs derived from both young and aged mice. For young BMSCs isolated from 6‐week‐old mice, the results of CCK‐8, cell cycle/apoptosis assay, and EdU assay indicated that APR had no significant impact on the cell proliferation or cell cycle distribution (Figure S3a–d). However, transwell migration assay demonstrated that APR significantly enhanced young BMSC migration (Figure 3a). In addition, ALP staining and Alizarin Red staining (ARS) assay showed an increase in ALP positive cells and mineralization nudes, indicating an enhanced osteogenic differentiation of young BMSCs (Figure 3b). qPCR and western blot analyses further confirmed the upregulation of osteogenic markers including ALP, OSX, RUNX2, COL1, OCN, and OPN during osteoblast differentiation following APR treatment (Figure 3c,d, Figure S3e).

To confirm the effect of activating ARs on aged BMSCs, cells were further isolated from 20‐month‐old mice. Beta‐galactosidase staining assay indicated a higher proportion of senescent cells in aged BMSCs compared to young BMSCs (Figure S4a,b). Consistently, aged BMSCs exhibited elevated gene and also protein expression levels of senescence‐associated genes including p53, p21, and p16 (Figure S4c,d). We next discovered that aged BMSC showed decreased non‐mitochondrial oxygen consumption, maximal mitochondrial aspiration and total ATP via conducting seahorse assay (Figure S4e), but increased reactive oxygen species (ROS) (Figure S4f). Immunofluorescence data of JC‐1 showed the mitochondrial membrane potential substantially descended in aged BMSCs (Figure S4g). Increased DNA damage marker, Histone H2A.X (Phospho Ser139), was found concentrated in the nuclear of aged BMSCs (Figure S4h). Similar to the findings in young BMSCs, no differences in cell viability or cell cycle distribution were observed in aged BMSCs after 24–48 h of APR treatment, as evidenced by CCK‐8, cell cycle/apoptosis, and EdU assays (Figure S5a–d). However, in contrast to young BMSCs, APR treatment at 5 μM significantly inhibited cell migration of aged BMSCs (Figure 3e) and decreased both ALP activity and mineralization after 5 and 14 days of osteoblast differentiation (Figure 3f). qPCR and western blot results further confirmed that osteogenic genes and proteins, including ALP, OSX, RUNX2, COL‐1, OCN, and OPN, were downregulated with APR treatment (Figure 3g,h, Figure S5e).

Additionally, senescence‐associated markers were assessed in both young and aged BMSCs after AdipoRon treatment as well. While no significant differences were observed in young BMSCs (Figure S6a), aged BMSCs showed significantly increased expression of *p21* and *p16*, suggesting that AR activation may accelerate cellular senescence in aged cells (Figure S6b). Furthermore, as increased BM adiposity is prevalent in older individuals, examining how AR activation differentially affects young and aged BMSCs on adipogenic is also essential. Intriguingly, our findings align with previous studies (Liu et al., 2021), demonstrating that APR significantly suppressed adipogenic differentiation in both young and aged BMSCs (Figure S7), and the increased BM adiposity may not be attributed to the increased APN actions during aging process.

### 2.5 AR1 played an essential role in osteogenesis under APR treatment in young BMSCs, while AR2 had a more significant effect in aged BMSCs

siRNA was employed to selectively knock down the expression of *Adipor1* and *Adipor2* in BMSCs, with knockdown efficiency verified by qPCR (Figure S8). In young BMSCs, ALP staining revealed that both *Adipor1*‐siRNA and *Adipor2*‐siRNA significantly reversed the APR‐induced increase in osteogenesis, but the *Adipor2*‐siRNA group exhibited higher ALP activity than the *Adipor1*‐siRNA group (Figure 3i,j). In contrast, in aged BMSCs, *Adipor2*‐siRNA almost completely counteracted the APR‐induced decrease in osteogenesis, whereas *Adipor1*‐siRNA only partially reversed this effect (Figure 3i,j).

Further analysis using qPCR and western blotting demonstrated that both *Adipor1*‐ and *Adipor2*‐siRNA could partially mitigate the APR‐induced upregulation of osteogenic markers such as ALP, Runx2, OSX, and COL1 in young BMSCs. Interestingly, the *Adipor1*‐siRNA group exhibited a more pronounced suppression of RUNX2 and OSX compared to the *Adipor2*‐siRNA group under APR treatment (Figure 3k, Figure S8a,b). Conversely, in aged BMSCs, knockdown of *Adipor1* had a limited effect on reversing the APR‐induced downregulation of these osteogenic markers. However, *Adipor2*‐siRNA dramatically rescued the suppressed expression of ALP, OSX, RUNX2, and COL1, highlighting its significant role in mediating APR effects in aged BMSCs (Figure 3k, Figure S8c,d).

### 2.6 Both AR1 and AR2 were involved in the inhibition of OC formation by APR in young and aged BMMs

The cell viability assays demonstrated that 48 h of APR treatment significantly inhibited the proliferation of both young and aged pre‐differentiated BMMs, with a more pronounced effect observed in young BMMs (Figure 3l). Cell cycle and EdU assay further revealed a significant reduction in the proportion of S‐phase cells in both age groups under APR treatment (Figure 3m), while the proportion of G2/M‐phase cells was higher in the APR‐treated group compared to the vehicle group (Figure 3l).

Following 7 days of OC differentiation with simultaneous APR treatment, TRAP staining results showed significant inhibition of OC formation in both young and aged BMMs (Figure 3n). Despite a dramatic decrease in OC size post‐APR treatment, similar to previous findings (Liu et al., 2021), the number of OCs remained unchanged between the vehicle and APR‐treated groups. qPCR and western blot analysis further confirmed the downregulation of osteoclastogenesis‐related genes and proteins, including CTSK, TRAP, and NFATc1, after 5 days of OC differentiation with 5 μM APR treatment in both young and aged BMMs (Figure 3o,p).

Subsequently, siRNA was used to knock down the expression of *Adipor*1 and *Adipor*2 in BMMs, with efficiency assessed by qPCR (Figure S9). At Day 5 post‐osteoclastogenic differentiation, both *Adipor1* and *Adipor2* knockdown rescued APR‐induced downregulation of TRAP in young BMMs. Notably, *Adipor2* knockdown had a more significant impact on the expression of CTSK and NFATc1 compared to Adipor1 knockdown (Figure 3q, Figure S9a,b). Similar patterns were observed in aged BMMs, where *Adipor1* knockdown partially reversed the decreased expression of TRAP induced by APR treatment, whereas *Adipor2* knockdown significantly upregulated the expression of TRAP, CTSK, and NFATc1 expression, which had been suppressed by APR treatment (Figure 3q, Figure S9c,d).

### 2.7 Different landscapes of cell–cell communications in BMSCs from young and aged mice

**FIGURE 4:**
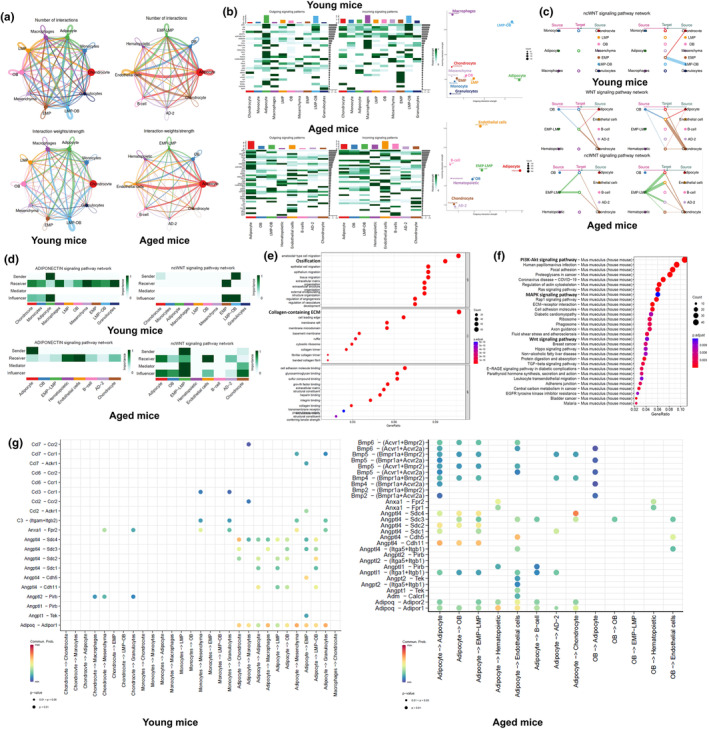
Cell–cell communications (CCCs) referenced by CellChat demonstrated notable alterations in receptors‐ligands‐mediated communications between 3‐ and 16‐month‐old mice. (a) Circle plots illustrate the number of interactions and interaction strength between bone marrow mesenchymal cells derived from 3‐ and 16‐month‐old mice. (b) Heatmap where the vertical axis represents cells sending or receiving signals, and the horizontal axis indicates the pathways involved in these communications. The color gradient in the heatmap signifies the strength of the signal, with accompanying bar graphs showing cumulative signal strengths along each axis. Additionally, a scatter plot details the intensity of outgoing and incoming interactions across a two‐dimensional manifold, where circle sizes indicate the number of significantly expressed receptor‐ligand pathways across different cell populations. (c) Hierarchical plot show the inferred intercellular communication network for Wnt signaling. Solid and open circles represent source and target cell types, respectively. Circle sizes are proportional to the number of cells in each cell type. (d) Heatmap presents the relative importance of each cell type as sender, receiver, mediator, and influencer, based on four network centrality measures specific to Adiponectin and Wnt signaling pathways. (e) Dot plots illustrate the top 10 significant GO enriched functions differing between 3‐ and 16‐month‐old mice (EMP‐LMP). The *x*‐axis shows the geneset ratio, while the y‐axis displays the geneset function. Dot colors correspond to adjusted *p*‐values, and sizes indicate the GeneRatio, increasing with the ratio. (f) The top 30 significant differential KEGG pathway enrichments in EMP‐LMP between 3‐ and 16‐month‐old mice are shown as a dot plot. The geneset ratio is plotted on the *x*‐axis, with enriched pathways on the y‐axis. Dot colors and sizes reflect adjusted P‐values and the GeneRatio, respectively. (g) Comparison of integral signal with superimposed input and output among bone marrow mesenchymal cells derived from 3‐ and 16‐month‐old mice. Dot colors indicate communication probabilities, and sizes represent computed *p*‐values from a one‐sided permutation test. Empty spaces denote a zero communication probability.

To further explore whether the distinct biological phenomena observed above might be attributed to variations in cell–cell communications (CCCs) among BMSCs between young and aged mice, we re‐analyzed previously generated single‐cell RNA sequencing data and performed CellChat analysis. This approach allowed us to investigate the characteristics of CCCs in BMSCs derived from young and aged mice. We analyzed the number and strength of interactions among different subpopulations of BMSCs, comparing these metrics between young and aged mice at an overall level (Figure 4a), This analysis revealed enhanced communication between adipocytes and mesenchymal progenitor cells in the BM of aged mice. We further explored the cell types that contribute to changes in CCCs patterns. By analyzing the strength of incoming and outgoing interactions, we identified significant changes in the signal‐sending and ‐receiving cell populations between the two groups, particularly in adipocytes, mesenchymal progenitor cells, and OB (Figure 4b). Hierarchical plots indicate that chondrocytes and OB are likely sources of non‐canonical Wnt signaling in young mice, targeting mesenchymal progenitor cells. In contrast, adipocytes in aged mice emerge as major sources of both non‐canonical and canonical Wnt signaling, targeting multiple cell types, including mesenchymal progenitor cells, OB, and adipocytes, each with varying strengths (Figure 4c). In addition to identifying the sender and receiver roles in Wnt signaling, we also determined which cell types act as mediators and influencers in Wnt and adiponectin signaling‐mediated intercellular communications. This was based on the relative importance of each cell type, quantified using an algorithm termed ‘centrality measure’ (Figure 4d). Moreover, GO and KEGG pathway enrichment analyses revealed significant differences in molecular functions and involved pathways, such as ossification, the PI3K‐AKT pathway, the MAPK pathway, and the Wnt pathway, in mesenchymal progenitors from young mice compared to those in aged mice (Figure 4e,f). Subsequently, we plotted and compared the ligand‐receptor pairs and the communication probabilities for each paired cell group between the two age groups, presenting an overall view of pathway variability. Notably, we observed significantly enhanced adiponectin‐AdipoR2 communication and reduced adiponectin‐AdipoR1 communication in aged mice across all different cell types compared to young mice (Figure 4g).

### 2.8 The AR1‐Wnt pathway is vital in young BMSCs subjected to APR treatment, while the AR2‐mTOR pathway is critical in aged BMSCs

**FIGURE 5:**
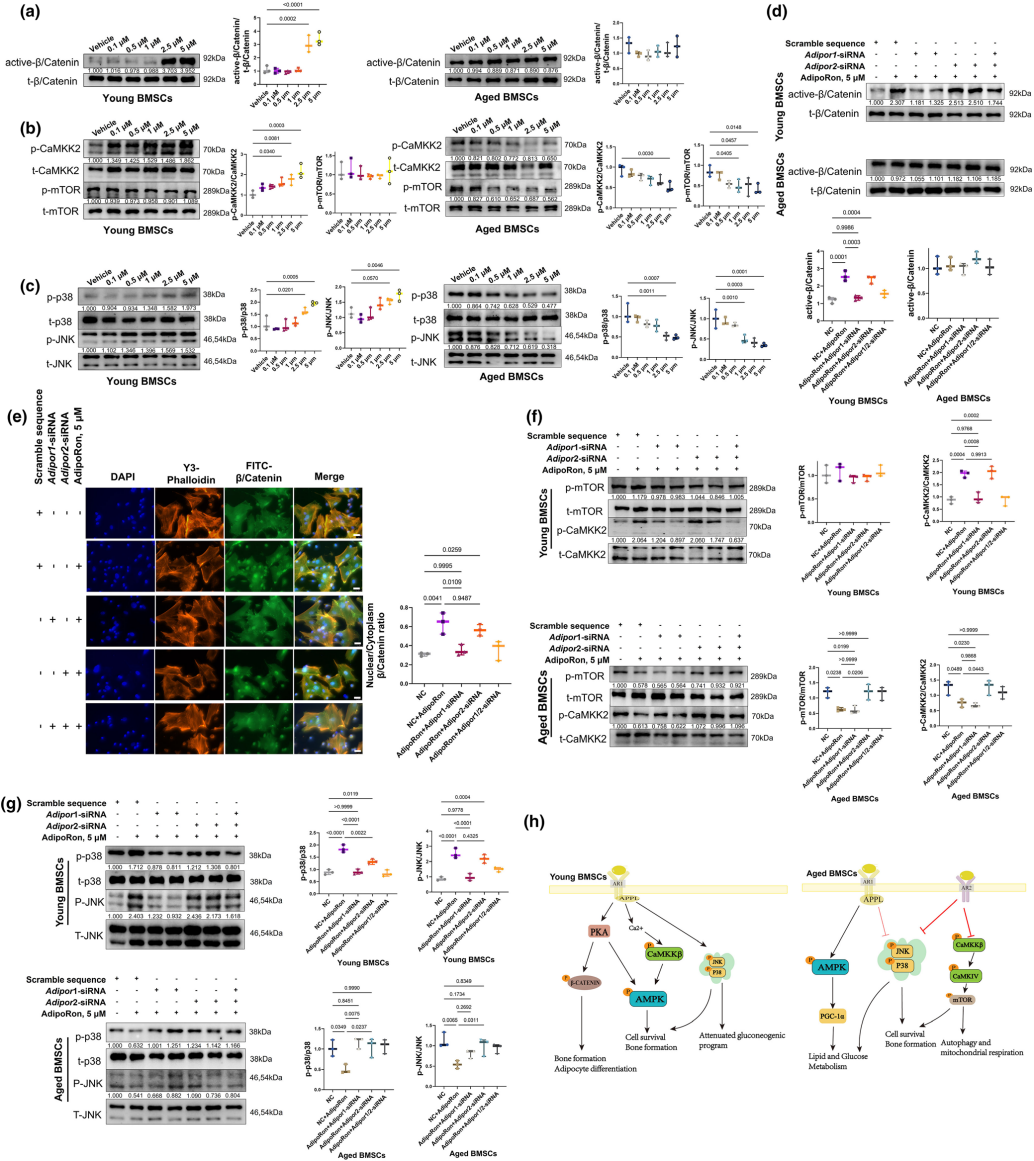
Wnt, mTOR, and MAPK pathway changes in pre‐differentiated young/aged BMSCs with APR treatment. (a) Wnt pathway changes and related quantification in young and aged BMSCs after 24 h APR treatment. Data shown as mean ± SD, *n* = 3 independent experiments; (b) p‐CaMKK2 and p‐mTOR changes and related quantification in young and aged BMSCs after 24 h APR treatment. Data shown as mean ± SD, *n* = 3 independent experiments; (c) p‐38 and p‐JNK changes and related quantification in young and aged BMSCs after 24 h APR treatment. Data shown as mean ± SD, *n* = 3 independent experiments; (d) Wnt pathway changes and related quantification in young and aged BMSCs after 24 h *Adipor1* or *Adipor2* siRNA treatment followed by 24 h APR treatment. Data shown as mean ± SD, *n* = 3 independent experiments; (e) β/Catenin nuclear translocation and related quantification in young BMSCs after 24 h *Adipor1* or *Adipor2* siRNA treatment followed by 12 h APR treatment. White bar: 25 μm. Data shown as mean ± SD, *n* = 3 independent experiments; Sale bar: 25 μm. (f) p‐CaMKK2 and p‐mTOR changes and related quantification in young and aged BMSCs after 24 h *Adipor1* or *Adipor2* siRNA treatment followed by 24 h APR treatment. Data shown as mean ± SD, *n* = 3 independent experiments; (g) MAPK pathway changes and related quantification in young and aged BMSCs after 24 h *Adipor1* or *Adipor2* siRNA treatment followed by 24 h APR treatment. Data shown as mean ± SD, *n* = 3 independent experiments. (h) Schematic diagram of involved signaling pathways in APR treated young and aged BMSCs.

Our study further examined the activation of the AMPK, MAPK, and Wnt signaling pathways, which are implicated in AR signaling. We observed increases in p‐AMPK, PGC‐1α, p‐ACC, and PPAR‐α protein levels in both young and aged pre‐differentiated BMSCs following APR treatment (Figure S10a,b). Specifically, in young pre‐differentiated BMSCs, APR treatment resulted in the upregulation of active β/Catenin, although no significant changes were noted in the p‐mTOR levels (Figure 5a,b). Additionally, levels of p‐p38 and p‐JNK were elevated in these cells treated with APR (Figure 5c). Conversely, in aged BMSCs, active β/Catenin did not show significant changes (Figure 5a), and there was a marked decrease in p‐CaMKK2 and p‐mTOR levels following APR treatment (Figure 5b). Levels of p‐JNK and p‐p38 were also slightly reduced in these aged BMSCs (Figure 5c).

To identify specific pathways related to different ARs between young and aged pre‐differentiated BMSCs, we further performed rescue experiments by knocking down *Adipor1* and *Adipor2* using siRNA (Figure S10c). In the Wnt pathway, *Adipor1* knockdown notably reduced the upregulation and nuclear translocation of β/Catenin in APR‐treated young BMSCs (Figure 5d,e), while no alterations were observed in aged BMSCs treated with APR or *Adipor1/2* siRNA. Moreover, *Adipor1* knockdown significantly rescued the level of p‐CaMKK2 in APR‐treated young BMSCs, while *Adipor2* knockdown had no effect. In contrast, *Adipor2* knockdown markedly countered the decreases in the p‐mTOR and p‐CaMKK2 levels in APR‐treated aged BMSCs (Figure 5f). Similarly, *Adipor1* knockdown significantly reversed the increases in the p‐p38 and p‐JNK levels in APR‐treated young BMSCs, with limited effects observed with *Adipor2* knockdown. Nonetheless, both *Adipor1* and *Adipor2* partly rescued the APR‐induced decreases in the p‐p38 and p‐JNK levels in aged BMSCs (Figure 5g).

In summary, both AR1‐AMPK and AR2‐PPAR‐α pathways were activated in young and aged BMSCs treated with APR. However, AR1‐MAPK and AR1‐Wnt signaling were predominantly activated in young BMSCs, while AR2‐MAPK and AR2‐mTOR signaling were inhibited in aged BMSCs (Figure 5h). These findings suggest that AR1‐Wnt and AR2‐mTOR signaling pathways may contribute to the differential responses to APR between young and aged BMSCs.

### 2.9 Similar AR signaling pathways were involved in APR‐treated young and aged BMMs

In this study, we also investigated the effects of APR on osteoclastogenesis‐related pathways including the NFκB, MAPK, and PI3K‐AKT pathways in BMMs. Unlike the varied responses observed in BMSCs, both young and aged pre‐differentiated BMMs exhibited similar responses to APR treatment. Specifically, there was a decrease in the levels of p‐IκBa, p‐p65, p‐JNK, p‐cJun, p‐PI3K, and p‐AKT levels in both young and aged BMMs after 24 h of APR treatment. Notably, only in aged BMMs, the p‐ERK level was significantly decreased after APR treatment (Figure S11a,b).

**FIGURE 6:**
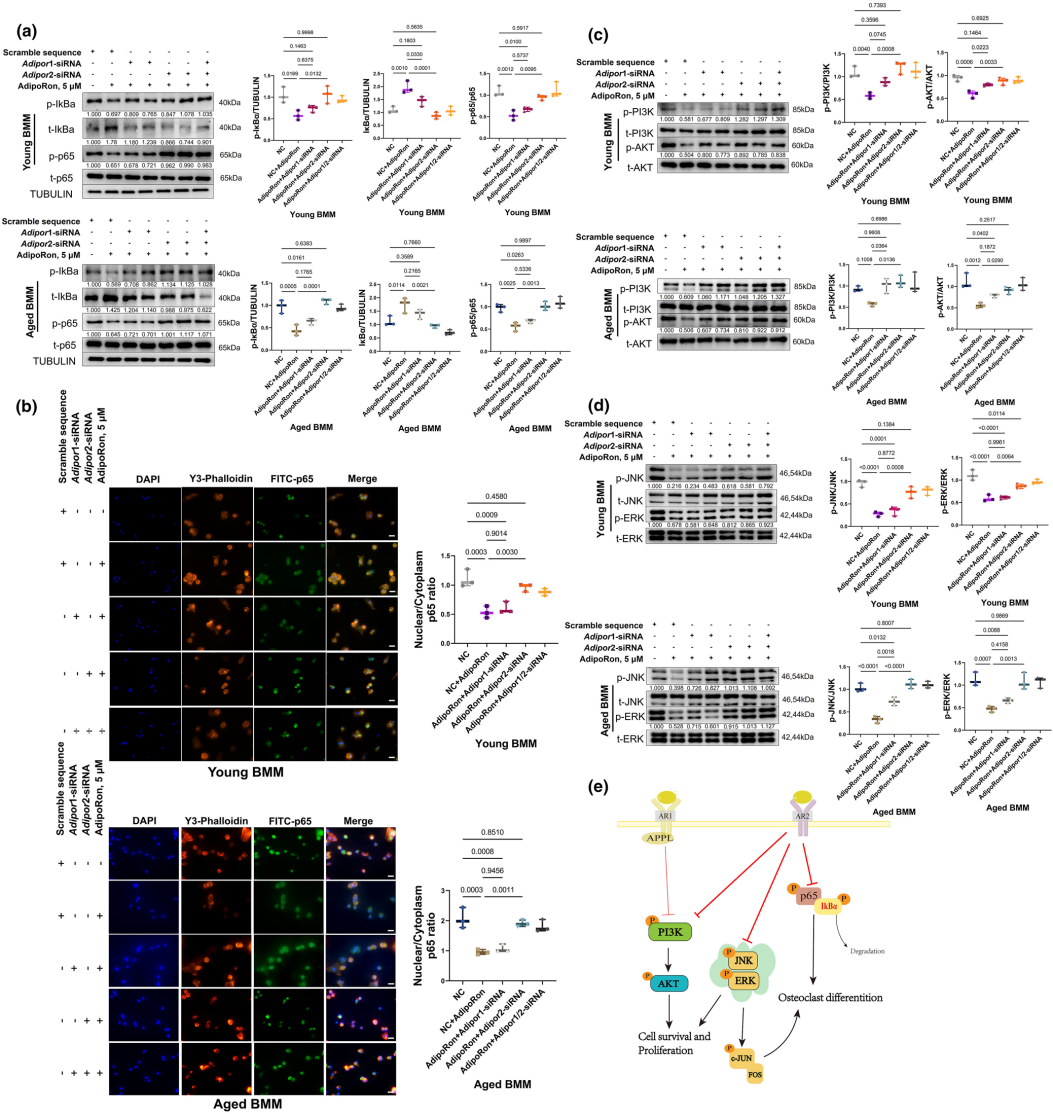
NFκB, AKT, and MAPK pathway changes in pre‐differentiated young/aged BMM with APR treatment. (a) NFκB pathway changes and related quantification in young and aged BMM after 24 h Adipor1 or Adipor2 siRNA treatment followed by 24 h APR treatment. Data shown as mean ± SD, *n* = 3 independent experiments; (b) p65 nuclear translocation and related quantification in young and aged BMM after 24 h Adipor1 or Adipor2 siRNA treatment followed by 24 h APR treatment. Data shown as mean ± SD, *n* = 3 independent experiments; Scale bar: 25 μm. (c) PI3K‐AKT pathway changes and related quantification in young and aged BMM after 24 h Adipor1 or Adipor2 siRNA treatment followed by 24 h APR treatment. Data shown as mean ± SD, *n* = 3 independent experiments; (d) MAPK pathway changes and related quantification in young and aged BMM after 24 h Adipor1 or Adipor2 siRNA treatment followed by 24 h APR treatment. Data shown as mean ± SD, *n* = 3 independent experiments; (e) Schematic diagram of involved signaling pathways in APR treated young and aged BMM.

To elucidate the roles of specific adiponectin receptors, we performed rescue experiments by knocking down *Adipor1* and *Adipor2* using siRNA (Figure S11c). Knockdown of *Adipor2* significantly reversed the APR‐induced decreases in the p‐IκBa and p‐p65 levels (Figure 6a), as well as the nuclear translocation of p65, in both young and aged BMMs (Figure 6b). In contrast, *Adipor1* knockdown showed limited effects on these markers (Figure 6a,b). Moreover, while either *Adipor1* knockdown or *Adipor2* knockdown could partially reverse the decreases in p‐PI3K and p‐AKT levels induced by APR, while *Adipor2* knockdown proved more effective (Figure 6c). Additionally, *Adipor2* knockdown almost completely abolished the APR‐induced decreases in the p‐ERK and p‐JNK levels, whereas *Adipor1* knockdown had limited effects (Figure 6d). These findings highlight the pivotal role of AR2 and its downstream pathways, including PI3K‐AKT, MAPK, and NFκB pathways, in mediating the inhibitory effects on osteoclastogenesis induced by APR treatment in both young and aged pre‐differentiated BMMs (Figure 6e).

## 3 DISCUSSION

APN, an adipocyte‐derived plasma protein that was discovered two decades ago, has gained attention as a potential therapeutic agent for osteoporosis and osteoporosis‐related bone fractures, primarily due to its roles in lipid metabolism and bone mineralization (Lenchik et al., 2003; Yokota et al., 2002). However, data on the in vivo, clinical, and in vitro effects of APN on bone regeneration and fracture repair are conflicting (Naot et al., 2017). These discrepancies may stem from the complexity of APN's circulating forms and the variability in its receptors, which likely result in diverse protein–receptor interactions and subsequent cellular responses.

A study by Lin found that 16‐month‐old transgenic mice overexpressing *Adipor1* had an increased BMD and reduced OC number, while younger mice aged 8 or 32 weeks did not exhibit these effects (Lin et al., 2014). Similarly, Tan reported that high circulating levels of APN adversely affect fracture risks and bone repair in overweight middle‐aged men, with no observable effects in younger individuals (Tan et al., 2014). Furthermore, Priya (Balasubramanian et al., 2022) reported that significantly higher expression of both *Adipor1* and *Adipor2* was observed in muscles from aged mice than in those from young mice, leading to a dramatically different cell response to APR. Our previous research corroborates these findings, showing that APR has opposite effects on the regulation of myogenesis and adipogenesis in muscle satellite cells derived from young and aged mice (Liu et al., 2023). These studies collectively suggest that the effects of APN on musculoskeletal regeneration and metabolism may vary substantially with age. However, the precise mechanisms underpinning the age‐related differences in AR signaling remain elusive and warrant further detailed investigation. Further mechanistic studies are needed to investigate how the aging process alters AR signaling within bone and systemically modulates bone regeneration and fracture repair.

In this study, we confirmed elevated levels of circulating APN in aged mice. Additionally, via scRNA‐seq and in vivo investigation, we observed age‐related variations in AR expression: aged mice exhibited lower AR1 but higher AR2 levels in BM compared to young mice. Specifically, young BMSCs demonstrated higher transcription level of *Adipor1* and lower transcription level of *Adipor2* than their aged counterparts, while young BMMs showed similar *Adipor1* but elevated *Adipor2* levels compared to aged BMMs. Aging appears to alter the transcriptional activity of *Adipor1*, *Adipor2*, and adiponectin within the BM niche. To investigate whether aging may alter the effect of APN on bone regeneration or fracture repair through the changes in *Adipo1r1/2* transcription, we established femur‐ and calvarial defect models in young and aged mice and assessed the impact of AR signaling on normal bone regeneration as well as bone defect repairing. Our findings align with our previous research indicating that APR accelerated bone formation and suppressed adipogenesis in young mice (Liu et al., 2021), and that sequential, controlled release of APR resulted in reduced levels of proinflammatory cytokines and increased alveolar bone repair in a periodontitis male mouse model (He et al., 2022). In the current study, we found that APR treatment enhanced bone regeneration, increased OCN‐positive cells, and enhanced osteoblast surface area while slightly reducing TRAP‐positive OCs in young mice, as opposed to vehicle treatment. In contrast, aged mice treated with APR exhibited inhibited trabecular and cortical bone regeneration, decreased OCN‐positive OB, and reduced TRAP activity compared to vehicle‐treated counterparts. This differential response in aged mice may be attributable to the distinct distribution of AR1 and AR2 in young versus aged BMSCs.

To elucidate the potential mechanism underlying the differential response to APR between young and aged mice, we isolated BMSCs and BMMs from different ages and treated them with APR. Consistent with our in vivo findings, APR treatment enhanced osteogenic differentiation and migration in young BMSCs, whereas it inhibited these processes in aged BMSCs. These data suggested that the higher serum APN level in aging individuals may lead to decreased BMSC osteogenesis, resulting in a concomitant decline in bone mass and strength. Intriguingly, APR inhibited adipogenic differentiation in both young and aged BMSCs, indicating that increased marrow adipogenesis observed during aging might not be directly related to higher circulating APN levels. Although no significant differences in the expression of cell senescence‐related markers, including *p21*, *p53*, and *p16,* were observed in young BMSCs after APR treatment, significantly higher expression of *p21* and *p16* was found in APR‐treated aged BMSCs, indicating that AR activation may accelerate senescence in aged BMSCs and thus inhibit their differentiation. However, this possibility seems inconsistent with the results reported by Sun et al. (2021), which showed that APR could rescue mitochondrial and cell senescence in aged skin by suppressing excessive division mediated by *Drp1*. As APR functions through AR activation, differences in receptor distribution between epidermal stem cells and BMSCs may account for these divergent effects.

Notably, a dramatic reduction in the levels of inflammatory cytokines, including TNF‐α, IL‐6, and IL‐1β was reported in aged epidermal stem cells, paralleling our findings that APR inhibited the canonical NFκB pathway in BMMs from both young and aged mice. This inhibition may subsequently lead to a downregulation of these cytokines in the macrophages. Furthermore, APR treatment was associated with slightly decreased cell proliferation and reduced osteoclastogenesis in BMMs across both age groups. These observations are in line with the findings of Wu et al., which demonstrated that APR can suppress osteoclastogenesis in BMMs by inhibiting the expression and nuclear translocation of NFATc1 (Wu et al., 2019).

To date, no study has explored the age‐dependent roles of different ARs and their downstream pathways in bone tissues or associated cells. Our *Adipor1/2* knockdown experiments revealed distinct responses between young and aged BMSCs. Specifically, *Adipor1* knockdown nearly reversed the increase in osteogenic differentiation seen in APR‐treated young BMSCs, while *Adipor2* knockdown was more effective in counteracting the APR‐induced inhibition of osteogenic differentiation in aged BMSCs. Additionally, siRNA targeting both *Adipor1* and *Adipor2* partially rescued the APR‐induced inhibition of osteoclastogenesis in both young and aged BMMs. These results suggest that in young BMSCs, AR signaling enhances osteogenesis predominantly through AR1 activation, whereas in aged BMSCs, it reduces osteogenesis primarily via AR2 activation. Existing evidence indicates that both AR1 and AR2 are involved in energy and bone metabolism but have opposing effects (Brochu‐Gaudreau et al., 2010). AR1 deficient mice exhibit a decreased energy expenditure and gradually become obese and glucose intolerant. In contrast, AR2 deficient mice exhibit an increased energy expenditure, and most of them are lean and resistant to high‐fat diet‐induced obesity (Ding et al., 2013). Given that aged BMSCs had significantly higher AR2 expression and lower AR1 expression than young BMSCs, we hypothesized that aging may modulate AR signaling based on the differential distribution of these receptors.

Several signaling pathways, including AMPK (Akimoto et al., 2018; Chen et al., 2015; Deepa & Dong, 2009; Fairaq et al., 2017; Okada‐Iwabu et al., 2013; Sara Malih, 2015; Wang et al., 2017), MAPK (Akimoto et al., 2018; Deepa & Dong, 2009; Fairaq et al., 2017; Lee et al., 2009; Luo et al., 2005), AKT (Akimoto et al., 2018; Sara Malih, 2015; Tu et al., 2011), and NFκB (Huang et al., 2010) pathways, are recognized as downstream of AR signaling. In this study, we investigated the potential mechanisms linking aging and APN actions by detecting changes in these pathways following APR treatment in young and aged cells. In young BMSCs, we observed activation of the pathways downstream of AR1, including the Wnt pathway and the JNK, p38, and CaMKK2/AMPK/PGC‐1α pathways, while AR2‐PPARα signaling was modestly upregulated. Conversely, in aged BMSCs, although the AR1‐AMPK/PGC‐1α pathway and AR2‐PPAR‐α signaling were activated, the CaMKK2/mTOR, JNK, and p38 pathways were inhibited as the downstream of AR2 (Figure 5h). These observations suggested that AR2 and its downstream pathways, specifically CaMKK2/mTOR and MAPK, may contribute to the decreased osteogenic differentiation observed with AR activation in aged BMSCs. Additionally, both young and aged BMMs displayed similar changes in AR signaling pathways in response to APR treatment, with inhibition of the JNK, ERK, PI3K/AKT, and NFκB pathways, and AR2 appeared to be the predominant receptor regulating these pathways (Figure 6e).

Despite the findings mentioned above, our study has a few limitations. First, since sex plays a significant role in adiponectin function, we only used male mice to minimize the influence of estrogen fluctuations on aging‐induced osteoporosis. Further studies are needed to explore the effects of sex on AdipoRon treatment. Second, while we re‐analyzed single‐cell RNA‐seq data from BM cells in young and aged mice without AdipoRon treatment, it would be more informative to include single‐cell sequencing data from mice treated with AdipoRon.

In conclusion, our study demonstrated both a pro‐osteogenic and anti‐osteoclastogenic effect of AR activation in young mice‐derived BMSCs and BMMs. Conversely, aged mice displayed impaired bone repair following APR treatment. Specifically, BMSCs derived from aged mice showed reduced migration and osteogenic differentiation under APR treatment, which may be attributed to enhanced cellular senescence driven by elevated AR2 expression and the subsequent activation of downstream mTOR pathways. On balance, these results suggest that AR signaling may promote bone regeneration and limit bone resorption in young individuals, but these effects become dysregulated in bones during the aging process. Increased APN levels, or differential *Adipor* transcriptional activity, and altered distribution of these receptors, along with subsequent activation of downstream pathways, pathologically tip the balance to favor delayed bone defect repair. Given that APR is a promising drug for the treatment of diabetes, it is essential to note the potential increased risk of bone fracture or delayed bone repair when applying APR to treat aging and diabetic patients.

## 4 MATERIALS AND METHODS

### 4.1 Reagents

AdipoRon was purchased from Selleck Chemicals, China. Small interfering RNAs (siRNAs) were designed by and purchased from GenePharma, China. Two siRNAs for each target transcript were used in this study.

### 4.2 Mouse bone defect model and APR treatment

All animal research was approved by the Animal Research Committee of Sichuan University (Chengdu, China) and followed all relevant guidelines and regulations. In total, 32 3‐ and 32 20‐month‐old C57BL/6 mice were used in this study. To omit the effect of estrogen, we used only male mice for this study. Based on the bone defect models (skull and femur defects) and treatment (vehicle and 50 mg/kg AdipoRon), all mice were allocated to different groups using the random number table method, and 8 mice were used per group. Mice were housed in polycarbonate cages on sterilized wooden bedding and maintained under 14:10 h light: dark cycles. Three mice from each age group were sacrificed before surgery, and the femora were fixed in 4% paraformaldehyde solution. In the femur defect group, a 1‐mm‐in‐diameter monolayer‐cortical bone defect (1 mm deep) was created 0.5 mm away from the growth plate on the distal side of the femur using a dental engine (Wallingford, USA). In the skull defect group, two 1‐mm‐in‐diameter double‐layer cortical bone defects were made 1 mm away from the cranial sagittal suture on both sides. Isoflurane was used for anesthesia before the surgery.

After surgery, APR diluted in corn oil was orally gavaged (50 mg/kg body weight) daily according to previously published studies (Balasubramanian et al., 2022; Liu et al., 2021; Okada‐Iwabu et al., 2013), and the control group was fed an equal volume of corn oil. All mice were sacrificed with overdose narcotics 2 weeks after surgery. Femurs, skull samples, and other organs, including the heart, kidney, and liver, were fixed in 4% paraformaldehyde solution and then stored in 70% ethanol.

### 4.3 Micro‐CT scanning and histology staining

The femur samples from presurgery mice and the skull and femur samples from 2‐week posttreatment mice were scanned by micro‐CT (u‐CT80, SCANCO, Switzerland) as described previously (Yang et al., 2019) and were reported according to international guidelines (Bouxsein et al., 2010). In normal mice, the micro‐architecture of trabecular bone in the distal femur metaphysis, as well as cortical bone, was analyzed using a high‐resolution μCT system. In the femur, the region of interest for trabecular bone began anterior to the growth plate break and extended proximally. The cortical bone region of interest spanned starting from a point located at 55% of the total bone length distal to the femoral head. Trabecular thickness (Tb. Th), and cortical bone thickness (Ct. Th) were evaluated. In bone defect model, a full‐thickness skull bone defect (diameter: 1 mm) and a 1 mm‐thick femur bone defect (diameter: 1 mm) from the bone surface were chosen as the regions of interest (ROI). Trabecular bone was segmented from soft tissue using a mineral density threshold of 375 mg HA/cm^3^, and cortical bone was segmented using a threshold of 700 mg HA/cm^3^. Total bone volume ratio (BV/TV), bone mineral density (BMD), trabecular separation (Tb. Sp), trabecular number (Tb. N), Tb. Th, and Ct. Th were evaluated.

After micro‐CT analysis, the skull and femur samples were processed for histology, as we previously reported (Yang et al., 2019). Bone tissue sections were subjected to tartrate‐resistant acid phosphatase (TRAP) staining and immunohistochemical staining with an anti‐osteocalcin antibody. TRAP‐positive cells, osteoblast surface, and bone surface were quantified for each section by Image‐Pro Plug software (version 6.0), and the region of interest (ROI) was restricted to the bone defect area (1 mm in diameter and 1 mm in depth). Cellular data on trabecular bone were represented by OC number (N. Oc/B. Pm) and osteoblast surface (Ob. S/BS) were measured. Potential drug toxicity was evaluated via H&E staining of heart, kidney, and liver tissues (data not shown).

### 4.4 Bone histomorphometry

Five 3‐month‐old and five 20‐month‐old C57BL/6 mice were used in this experiment. Mice received intraperitoneal injections of 20 mg/kg calcein (Sigma, St. Louis, MO) 9 and 2 days prior to sacrifice. Dynamic histomorphometric analyses were conducted on 3‐ and 20‐month‐old mice 2 weeks following treatment with APR and vehicle, as previously described (Le et al., 2021). Femurs were analyzed using standard nomenclature. Briefly, dynamic data from unstained sections were obtained using a microscope under fluorescent settings. The mineral apposition rate (MAR) and bone formation rates (BFR, MS/BS) were measured in the periosteum of cortical bone.

### 4.5 Single cell RNA‐seq, bulk RNA‐seq, and ATAC‐seq analysis

Analyses of single cell RNA‐seq (scRNA‐seq) data, bulk RNA‐seq and ATAC‐seq were carried out in the R package Seurat. For single cell transcriptomic profiling of Col2+ BM mesenchymal lineage cells between 3‐ and 20‐month‐old mice, datasets from GSE145477 were used (Zhong et al., 2020). Cluster specific markers for each cluster, relative to the included population, were conducted using the FindMarkers to identify differentially expressed genes (DEGs). Sub‐clustering was performed by isolating the mesenchymal lineage clusters identified from the remaining BM cells using known marker genes, followed by reanalysis. T‐distributed stochastic neighbor embedding (tSNE) plots were used to visualize the expression level of *Adipor1*, *Adipor2*, and *Adipoq* among different cell clusters. GO terms and KEGG pathway enrichment of DEGs between groups, were identified using the database for annotation, visualization and integrated discovery (DAVID). For determination of the potential interactions between different cell clusters, we performed cell–cell communication analysis using the R package CellChat.

For bulk RNA‐seq and ATAC‐seq, isolated SCA‐1+, CD140a^+^ and CD45‐, TERR‐119‐ BMSCs from 3‐ to 20‐month‐old mice were used for sequencing, and the datasets were obtained from GSE143580 (Pouikli et al., 2021). The sequenced reads of RNA‐seq dataset were processed using zUMIs (v.2.2.1) with STAR, Samtools and featureCounts from Rsubreads. The reads were mapped to the mouse genome (mm10) with the ensemble annotation v. GRCm38.91. The generated count matrix was further analyzed using R. The fastq files of ATAC‐seq reads were mapped to the mouse genome (mm10) using Bowtie2 and the resulting BAM files were sorted, indexed using Samtools. The MACS2 was used to call peaks, using a *p*‐value threshold of 0.01 and default settings. HOMER was used for peak annotation, and peak differences between young and aged mice were analyzed using the R DESeq2 package. The differential gene expression analysis between young and aged mice was carried out, and the obtained sets of overlapping genes from both RNA‐seq and ATAC‐seq were further analyzed, for example through GO and KEGG enrichment analysis.

### 4.6 Cell culture, osteogenic and adipogenic differentiation, and osteoclastogenesis induction

For osteogenic differentiation, BMSCs were routinely cultured as we described previously (Xu et al., 2018; Yang et al., 2019). Briefly, tibiae and femora were cut and spun by centrifugation, and cells were then resuspended in a complete culture medium. BMSCs were purified by passaging, and passage 2 cells were used in all experiments. According to previous studies that showed no significant difference in the expression of cell surface markers in BMSCs with age (Fossett et al., 2012; Mareschi et al., 2006), CD29, CD 90, CD34, and CD45 markers were used for cell flow cytometric analysis to demonstrate that a consistent BMSC population was being used in each experiment. Senescence‐associated analysis was performed using β‐galactosidase staining. Cells were treated with or without 5 μM APR combined with osteogenic medium, which consisted of complete αMEM (αMEM, 5% fetal bovine serum, and 1% penicillin/streptomycin), 50 μg/mL ascorbic acid, and 8 mM beta‐glycerophosphate (both were purchased from Sigma, St. Louis, MO) simultaneously for 5 days to perform alkaline phosphatase (ALP) staining and ALP quantitation or for 14 days to conduct Alizarin red staining. AR‐stained cells were destained with 10% cetylpyridinium chloride for 1 h, and their absorbance was measured at 570 nm for quantitation.

For adipogenic differentiation, BMSCs were differentiated into adipocytes once cells reached 100% confluency. Adipogenic differentiation was initiated with the addition of adipogenic reagents, including 0.5 mM IBMX, 1 μM dexamethasone, 10 μg/mL insulin, and 15 μM rosiglitazone, to the Base DMEM [DMEM High Glucose (Life Technologies, Carlsbad, CA), 10% fetal bovine serum (FBS) (VWR, Radnor, PA), 1% penicillin/streptomycin (PS) (Life Technologies, Carlsbad, CA)]. Nine days after adipocyte induction, cells were fixed with 10% neutral buffered formalin and were ready for Oil Red O (ORO) staining. RNA was extracted on Day 9 for real‐time PCR analysis.

For osteoclastogenesis, BM‐derived macrophages (BMMs) were isolated from the femora and tibiae of male C57BL/6 mice. After 48 h of adherent culture, the cells in suspension were collected and cultured with a conditioned medium containing recombinant M‐CSF (Peprotech, US) at 30 ng/mL. Osteoclastogenic differentiation was performed as we previously described by adding 30 ng/mL M‐CSF and 100 ng/mL RANKL (Luo et al., 2012). Passage 0 cells were used for further experiments. F4/80 and CD 68 were used as markers for flow cytometric analysis to demonstrate that a consistent BMM population was used in each experiment. TRAP staining was performed after 6 days of differentiation, with or without simultaneous 5 μM APR treatment, using a TRAP/ALP stain kit (Wako, Japan) according to the manufacturer's instructions. Related gene and protein expression levels were also detected at the same time points.

### 4.7 Cell proliferation assay

A cell counting kit (CCK‐8 kit, Dojindo, Japan), flow cytometric cell cycle analysis (Cell Cycle Detection Kit, KeyGen Biotech, China), and EdU incorporation assay (iClick EdU Andy Fluor 488 Imaging Kit, GeneCopoeia, US) were performed to analyze the effect of APR on the viability of all tested cells after 24 and 48 h of 5 μM APR treatment. EdU‐positive cells were observed by fluorescence microscopy (Olympus, Tokyo, Japan).

### 4.8 Cell migration assay

Transwell assays were used to investigate the cell migration ability of BMSCs. Cells were plated on the top chamber overnight, then the medium of the top chamber was changed to serum‐free medium, and the bottom was changed to 10% FBS medium with or without 5 μM APR. Eighteen hours later, inserts were washed with PBS, and the migratory cells were fixed in 4% formaldehyde, stained with 0.5% crystal violet, and counted in five fields using a light microscope.

### 4.9 RNA interference

Twenty‐four hours after seeding, RNA oligos were transfected into BMSCs or BMMs using EndoFectin Max (GeneCopoeia, US) according to the manufacturer's instructions, and the final concentration of siRNA was 5 nM. The transfection medium was changed to an osteogenic differentiation medium after 24 h of incubation. Cells were fixed and stained after 5 days of osteogenic differentiation or collected for further experiments after 6–24 h of treatment, with or without APR.

### 4.10 RNA extraction and quantitative reverse transcription polymerase chain reaction analysis

TRIzol lysis buffer (Takara, Japan) was used to extract total RNA from the treated cells, and reverse transcription and quantitative polymerase chain reaction (qPCR) was performed as described previously (Yang et al., 2019). The primers used for qPCR are listed in Table S1.

### 4.11 Immunoblotting and immunofluorescence

Immunoblots were performed as previously described (Yang et al., 2019). The antibodies used for immunoblotting are listed in Table S2. Each experiment was repeated with 3 independent experiments, and the most representative blots were chosen for presentation if similar results were observed. Quantitation of immunoblots was conducted via ImageJ software. BMSCs and BMMs were plated on an 8‐chamber slide (Sigma, US) for cell immunofluorescence. Anti‐β/Catenin rabbit monoclonal (Cell Signaling, US) or anti‐p65 rabbit monoclonal (Cell Signaling, US) antibodies and FITC‐labeled secondary antibodies were used for cell immunofluorescence. The cytoskeleton was stained with phalloidin‐rhodamine, and nuclei were counterstained with DAPI.

### 4.12 Elisa

The APN content in the serum of 3‐ and 20‐month‐old mice was detected using a mouse APN ELISA kit (R&D, US) according to the manufacturer's instructions.

### 4.13 Statistical analysis

All quantitative data are presented as box and whisker (median and range) plots. Statistical significance was assessed between 2 groups with a *t*‐test and among 3 or more groups with one‐way analysis of variance (ANOVA) following Dunnett's test using SPSS software (version: 17.0) and GraphPad Prism (version: 7.0). Values of *p* < 0.05 were considered to indicate significant differences.

## AUTHOR CONTRIBUTIONS

Hanghang Liu: Conceptualization, investigation, methodology, formal analysis, resources, validation, visualization, writing–original draft preparation. Qiucheng Zhao: Investigation, methodology, formal analysis, data curation, validation, visualization, writing–original draft preparation. Shibo Liu: Investigation, formal analysis, visualization, writing–original draft preparation. Bolun Li: Formal analysis, visualization. Zizhuo Zheng: Formal analysis, visualization. Yao Liu: Conceptualization, supervision, writing–review, and editing. Pei Hu: Conceptualization, supervision, writing–review, and editing. En Luo: Conceptualization, funding acquisition, supervision, writing–review, and editing.

## CONFLICT OF INTEREST STATEMENT

The authors declare no conflicts of interest.

## Supporting information


**Figure S1.** The quality control information of the included samples for single cell RNA‐seq.
**Figure S2.** Expression level of AR1 and AR2 in young and aged BMM.
**Figure S3.** APR treatment showed no effect on the proliferation of young BMSCs.
**Figure S4.** Cell characters for young and aged BMSCs.
**Figure S5.** APR treatment showed no effect on the proliferation of aged BMSCs.
**Figure S6.** AdipoRon promoted cell senescence in aged BMSCs but had no significant effect on young BMSCs.
**Figure S7.** APR treatment suppressed adipogenic differentiation of both young and aged BMSCs.
**Figure S8.** qPCR and western blot results for young and aged BMSC.
**Figure S9.** qPCR and western blot results for young and aged BMM.
**Figure S10.** Activated pathways in young and aged BMSCs with 24 h APR treatment and siRNA efficiency confirmation.
**Figure S11.** Activated pathways in young and aged BMM with 24 h APR treatment and siRNA efficiency confirmation.
**Table S1.** Sequence of primers.
**Table S2.** Antibody list.

## Data Availability

The data that support the findings of this study are available from the corresponding author upon reasonable request. The datasets for scRNA‐seq and bulk RNA‐seq + ATAC‐seq were obtained from GSE145477 and GSE143580, respectively.
