# Supplementary material for: Aging alters the effect of adiponectin receptor signaling on bone marrow‐derived mesenchymal stem cells
Source: Aging Cell. 2024 Oct 27;24(2):e14390. doi: 10.1111/acel.14390 (PMC11822658; doi:10.1111/acel.14390)
Supplement: Supplementary file 1 — Figure S1. The quality control information of the included samples for single cell RNA‐seq. Figure S2. Expression level of AR1 and AR2 in young and aged BMM. Figure S3. APR treatment showed no effect on the proliferation of young BMSCs. Figure S4. Cell characters for young and aged BMSCs. Figure S5. APR treatment showed no effect on the proliferation of aged BMSCs. Figure S6. AdipoRon promoted cell senescence in aged BMSCs but had no significant effect on young BMSCs. Figure S7. APR treatment suppressed adipogenic differentiation of both young and aged BMSCs. Figure S8. qPCR and western blot results for young and aged BMSC. Figure S9. qPCR and western blot results for young and aged BMM. Figure S10. Activated pathways in young and aged BMSCs with 24 h APR treatment and siRNA efficiency confirmation. Figure S11. Activated pathways in young and aged BMM with 24 h APR treatment and siRNA efficiency confirmation. Table S1. Sequence of primers. Table S2. Antibody list. [file ACEL-24-e14390-s001.docx]

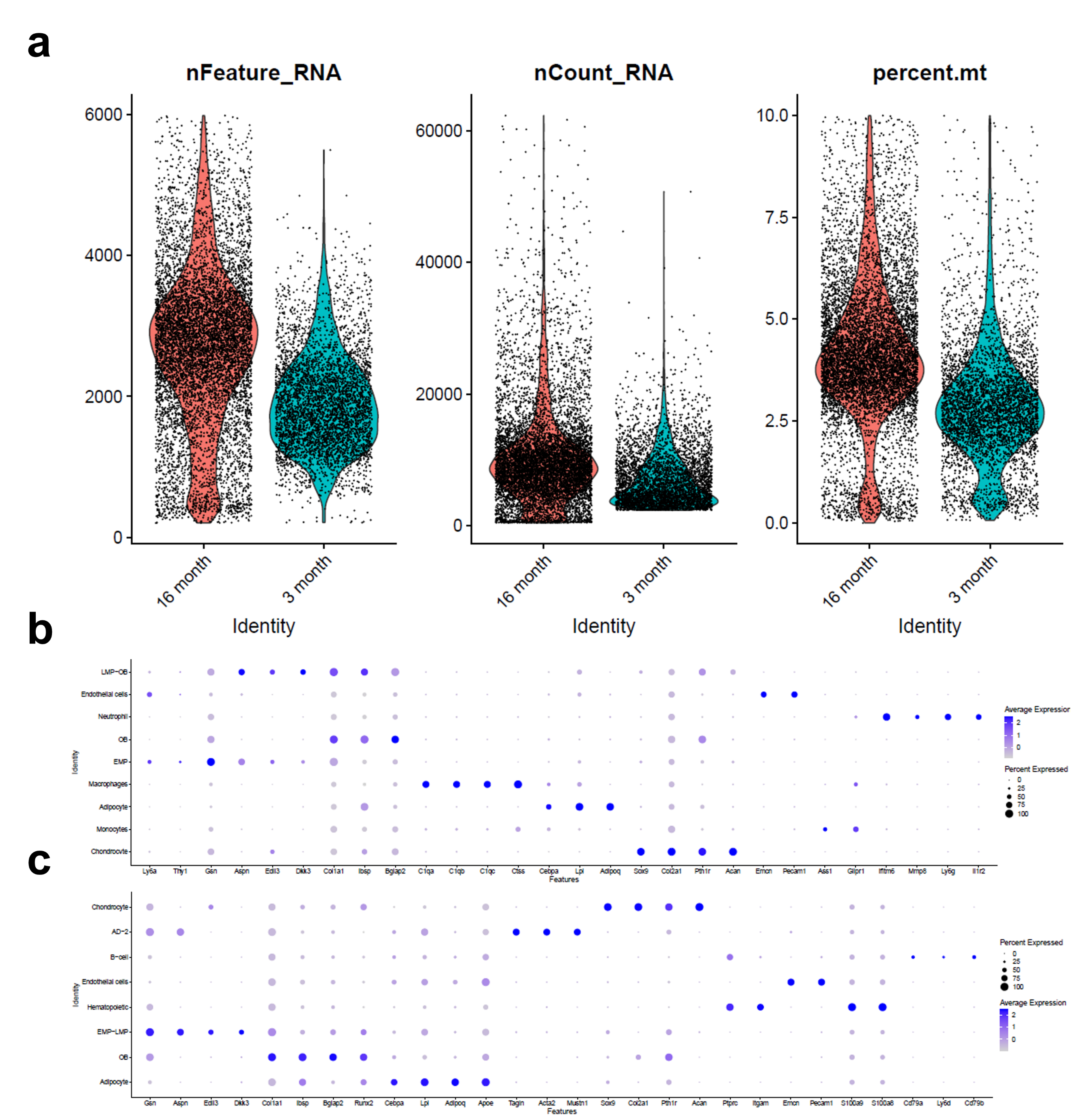


**Fig. S1** The quality control information of the included samples for single cell RNA-seq. **a** Violin plots showing number of genes, UMIs, and percent mitochondrial reads for all cells in the full dataset (nFeature_RNA > 200 & nFeature_RNA < 6000 & percent.mt < 10). **b, c** Dot plot showing expression of selected marker genes in each cell type for the Col2+ BMSCs from 3-month-old and 16-month-old mice, respectively. The size of the dot indicates the percentage of cells within a cell type in which that marker was detected, and its color indicates the average expression level.


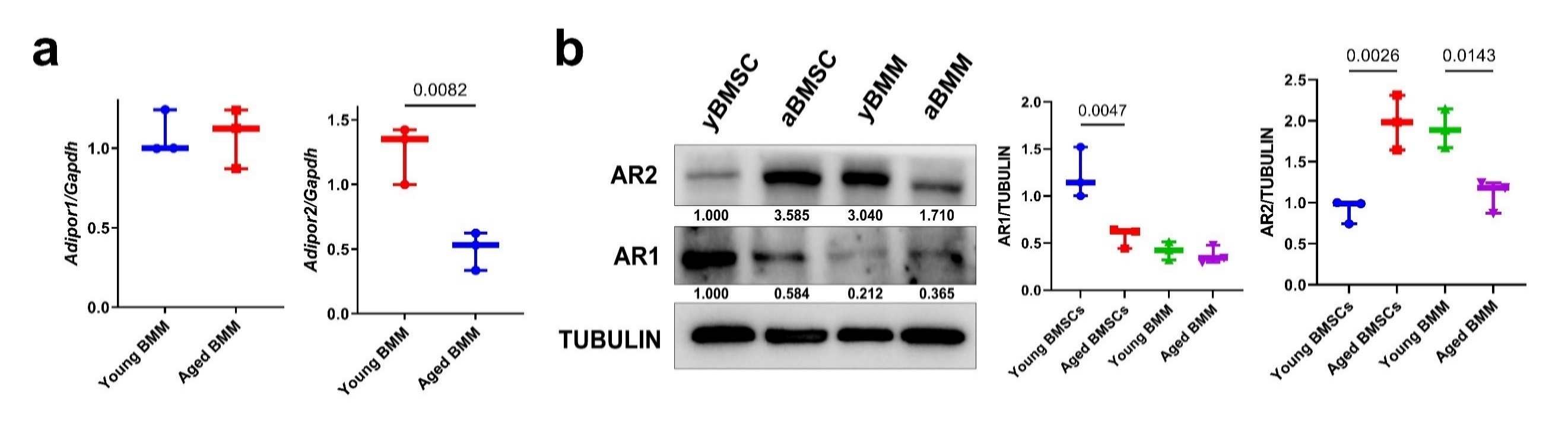


**Fig. S2** Expression level of AR1 and AR2 in young and aged BMM. **a** qPCR results of *Adipor1* and *Adipor2* in BMM derived from 6-week-old and 20-month-old male mice, n=3. **b** Immunoblot and related quantification results of AR1 and AR2 in BMSC and BMM derived from 6-week-old and 20-month-old male mice, n=3. yBMSCs, young BMSC; yBMM, young BMM; aBMSC, aged BMSCs; aBMM, aged BMM.


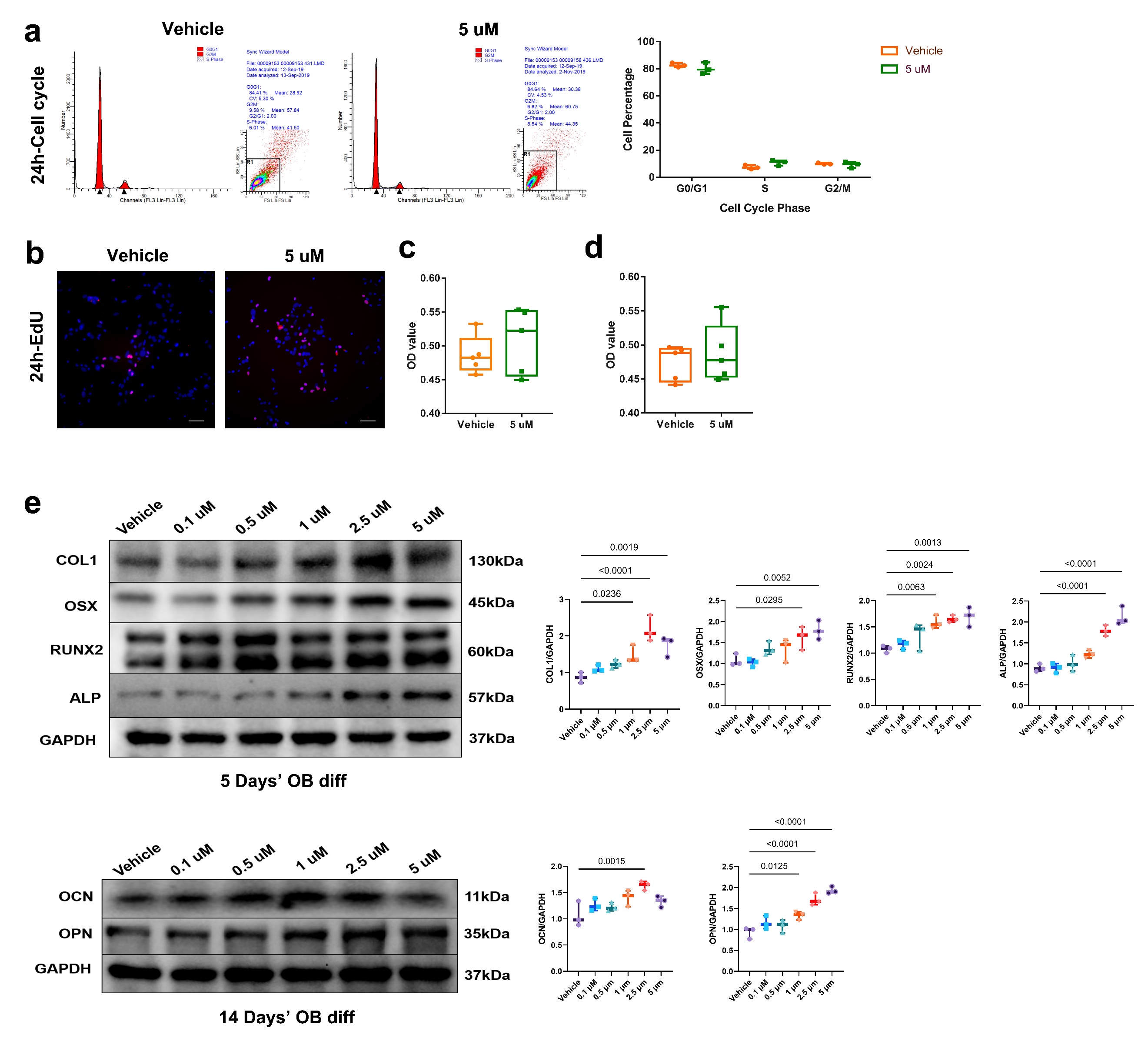


**Fig. S3**. APR treatment showed no effect on the proliferation of young BMSCs. **a** Cell cycle/apoptosis flow cytometry analysis of young BMSCs after 24 hours’ APR treatment and related quantitative analysis, n=3. **b** Representative pictures for EdU staining of young BMSCs after 24h APR treatment and 24h EdU incubation (with APR), white scale bar: 500 um. **c** CCK-8 assay of young BMSCs after 24 hours’ APR treatment, n=5. **d** CCK-8 assay of young BMSCs after 48 hours’ APR treatment, n=5. **e** Western blot and related quantification results of young BMSCs after 5- and 14-days’ OB differentiation, n=3.


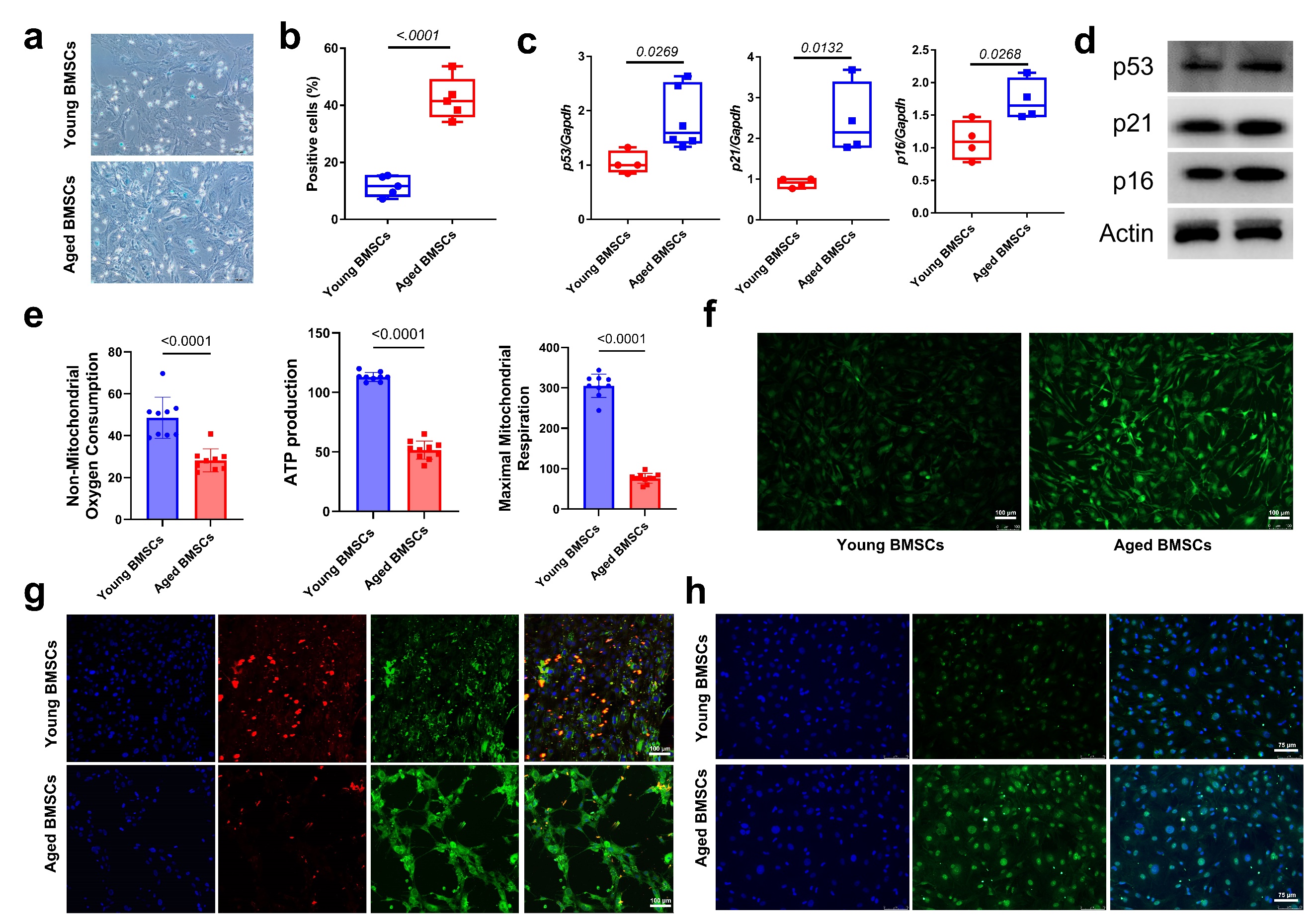


**Fig. S4.** Cell characters for young and aged BMSCs. **a,b** β-galactosidase staining results and related quantification for young and aged BMSCs, n=5; Scale bar: 100μm; **c** qPCR results of *p53*, *p21*, *p16* for young and aged BMSCs, n=6. **d** Western blot results of p53, p21, p16 for young and aged BMSCs. **e** Oxygen consumption and ATP production in young and aged BMSCs detected by seahorse assay. **f** Representative images of ROS concentration in young and aged BMSCs, Scale bars, 100 μm. **g** Representative images of immunofluorescence staining using JC-1 probes. Scale bars, 100 μm. **h** Representative images of immunofluorescence staining using Histone H2A.X antibody. Scale bars, 100 μm.


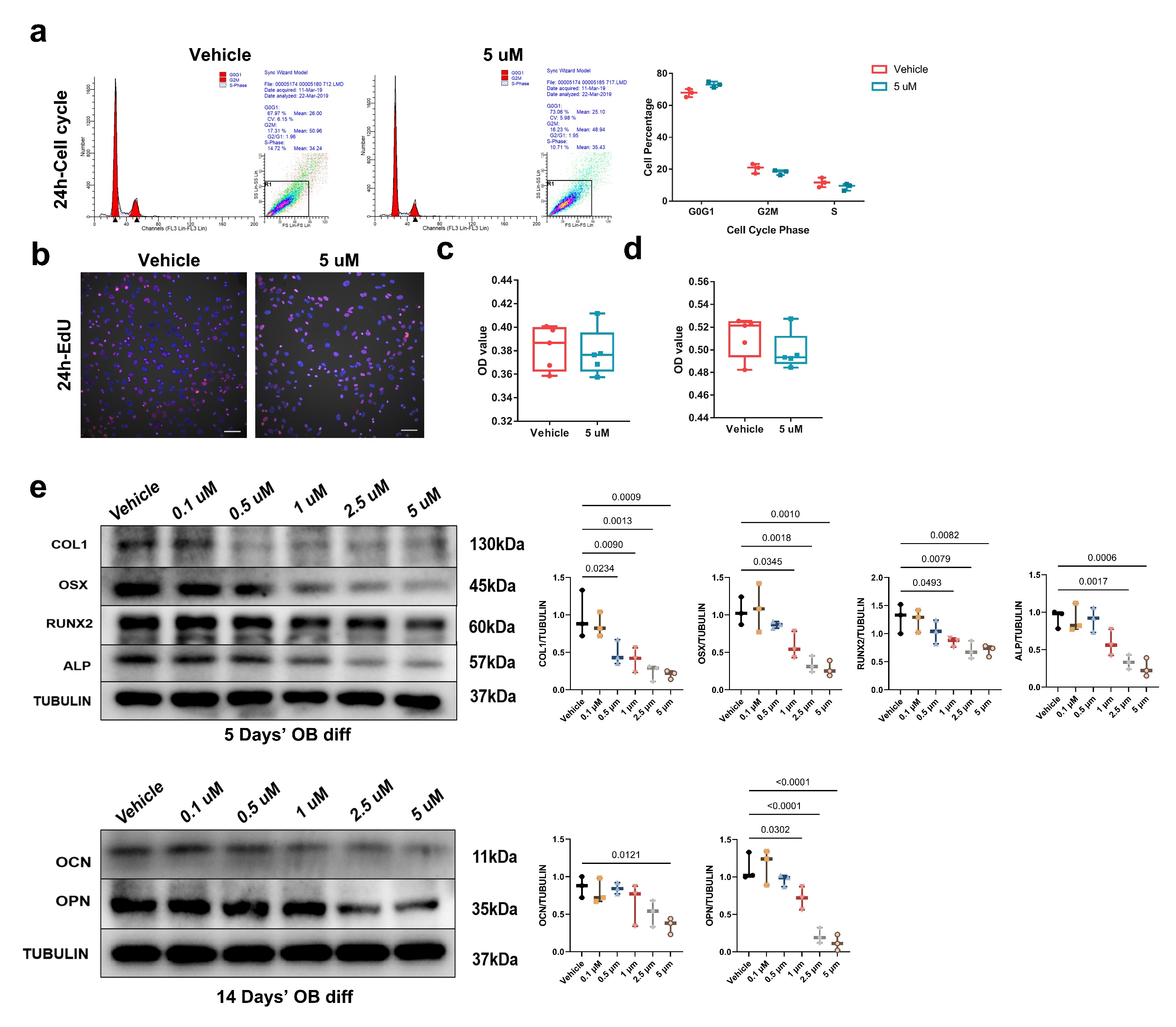


**Fig. S5**. APR treatment showed no effect on the proliferation of aged BMSCs. **a** Cell cycle/apoptosis flow cytometry analysis of aged BMSCs after 24 hours’ APR treatment and related quantitative analysis, n=3. **b** Representative pictures for EdU staining of aged BMSCs after 24h APR treatment and 24h EdU incubation (with APR), white scale bar: 500 um. **c** CCK-8 assay of aged BMSCs after 24 hours’ APR treatment, n=5. **d** CCK-8 assay of aged BMSCs after 48 hours’ APR treatment, n=5. **e** Western blot results of young BMSCs after 5 and 14 days’ OB differentiation.

**
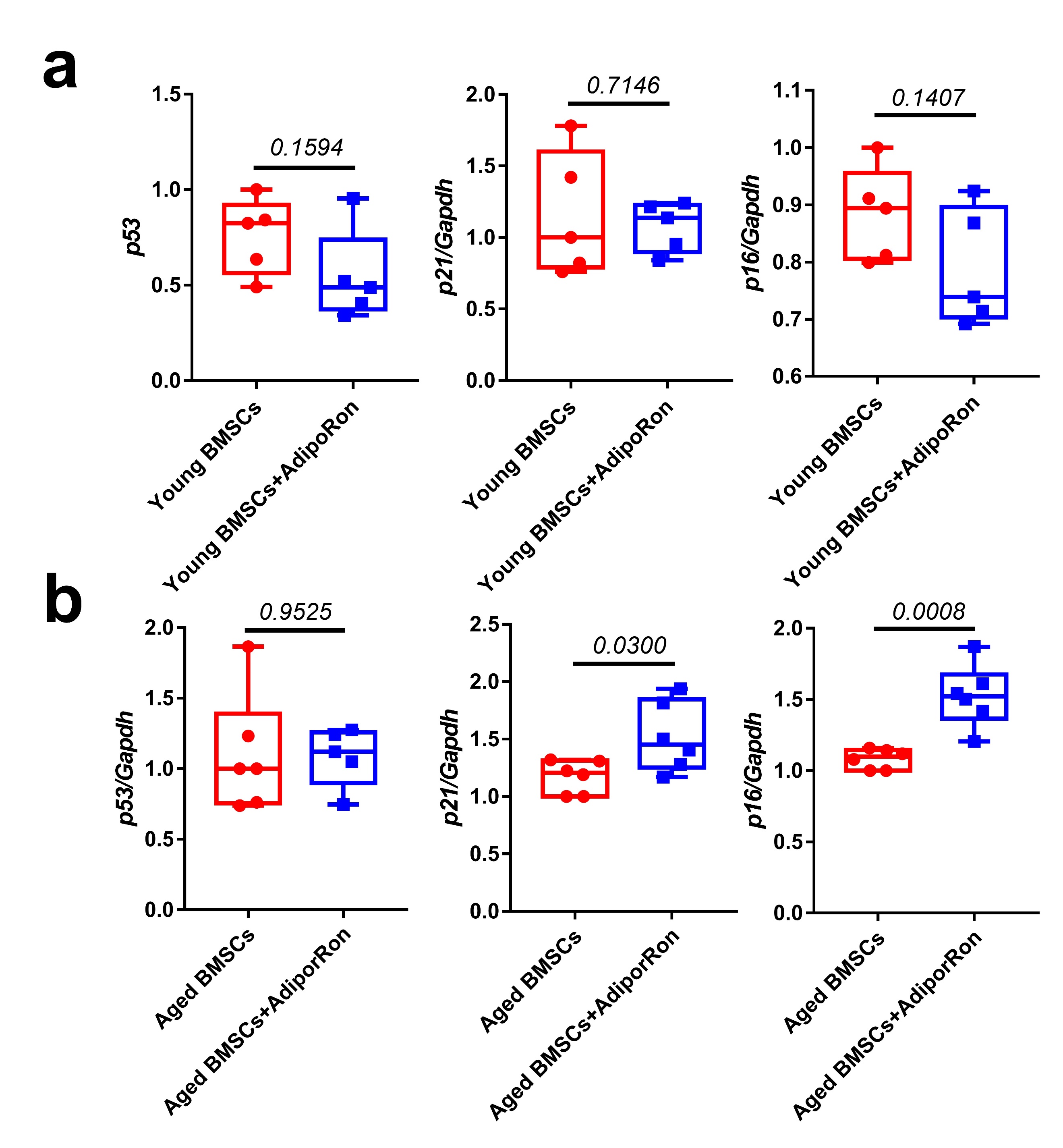
**

**Fig. S6** AdipoRon promoted cell senescence in aged BMSCs but had no significant effect on young BMSCs. **a** qPCR results of *p53*, *p21*, *p16* for young BMSCs treated with AdipoRon for 48 hours, n=5. **b** qPCR results of *p53*, *p21*, *p16* for aged BMSCs treated with AdipoRon for 48 hours, n=5.


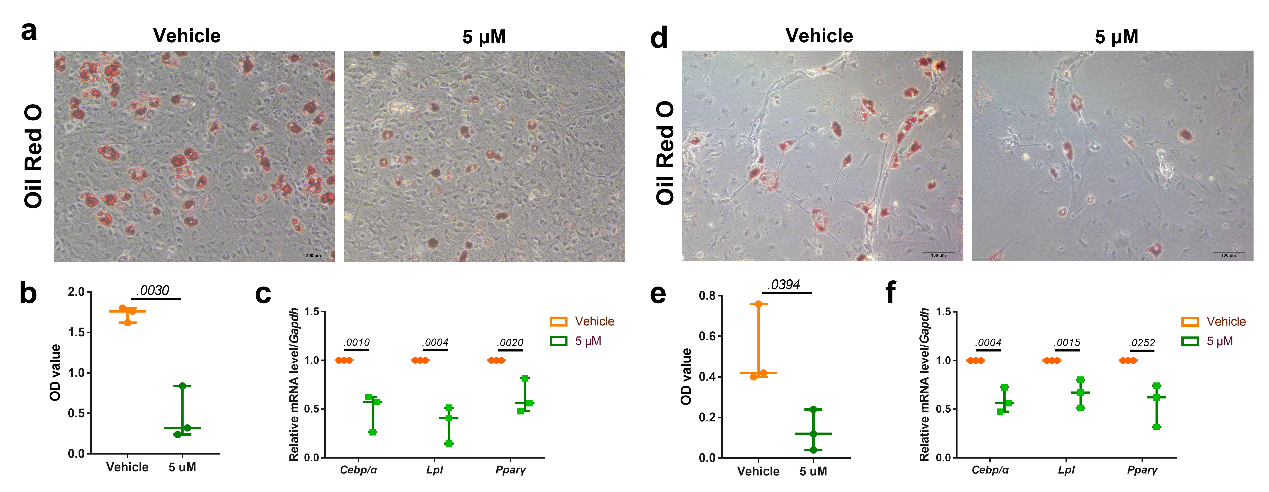


**Fig. S7**. APR treatment suppressed adipogenic differentiation of both young and aged BMSCs. **a, b** Representative images of ORO staining of young BMSCs after 9 days’ adipogenic differentiation and related quantification, n=3. **c** qPCR results from young BMSCs after 9 days’ adipogenic differentiation, n=3. **d, e** Representative images of ORO staining of aged BMSCs after 9 days’ adipogenic differentiation and related quantification, n=3. **c** qPCR results from aged BMSCs after 9 days’ adipogenic differentiation, n=3.


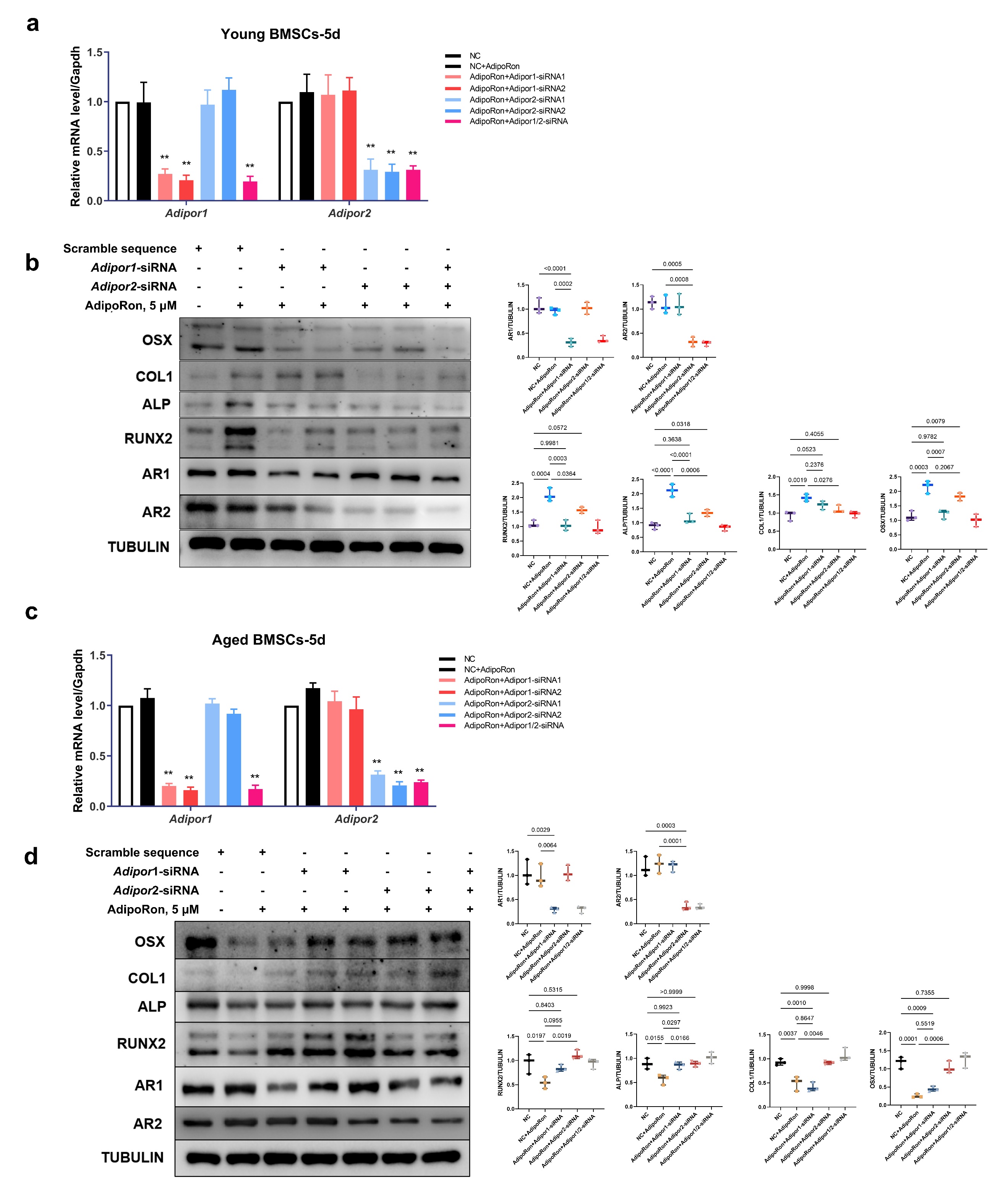


**Fig. S8.** qPCR and western blot results for young and aged BMSC. **a** qPCR results of *Adipor1* and *Adipor2* for young BMSCs after 5 days’ OB differentiation followed by 24 hours’ siRNA treatment, n=3. Data shown as mean ± SD. *p<.05 vs. NC+AdipoRon group; **p<.01 vs. NC+AdipoRon group. **b** Immunoblot results of osteogenseis related proteins for young BMSCs after 5 days’ OB differentiation followed by 24 hours’ siRNA treatment. n=3. **c** qPCR results of *Adipor1* and *Adipor2* for aged BMSCs after 5 days’ OB differentiation followed by 24 hours’ siRNA treatment, n=3. Data shown as mean ± SD. *p<.05 vs. NC+AdipoRon group; **p<.01 vs. NC+AdipoRon group. **d** Immunoblot results of osteogenseis related proteins for aged BMSCs after 5 days’ OB differentiation followed by 24 hours’ siRNA treatment. n=3.


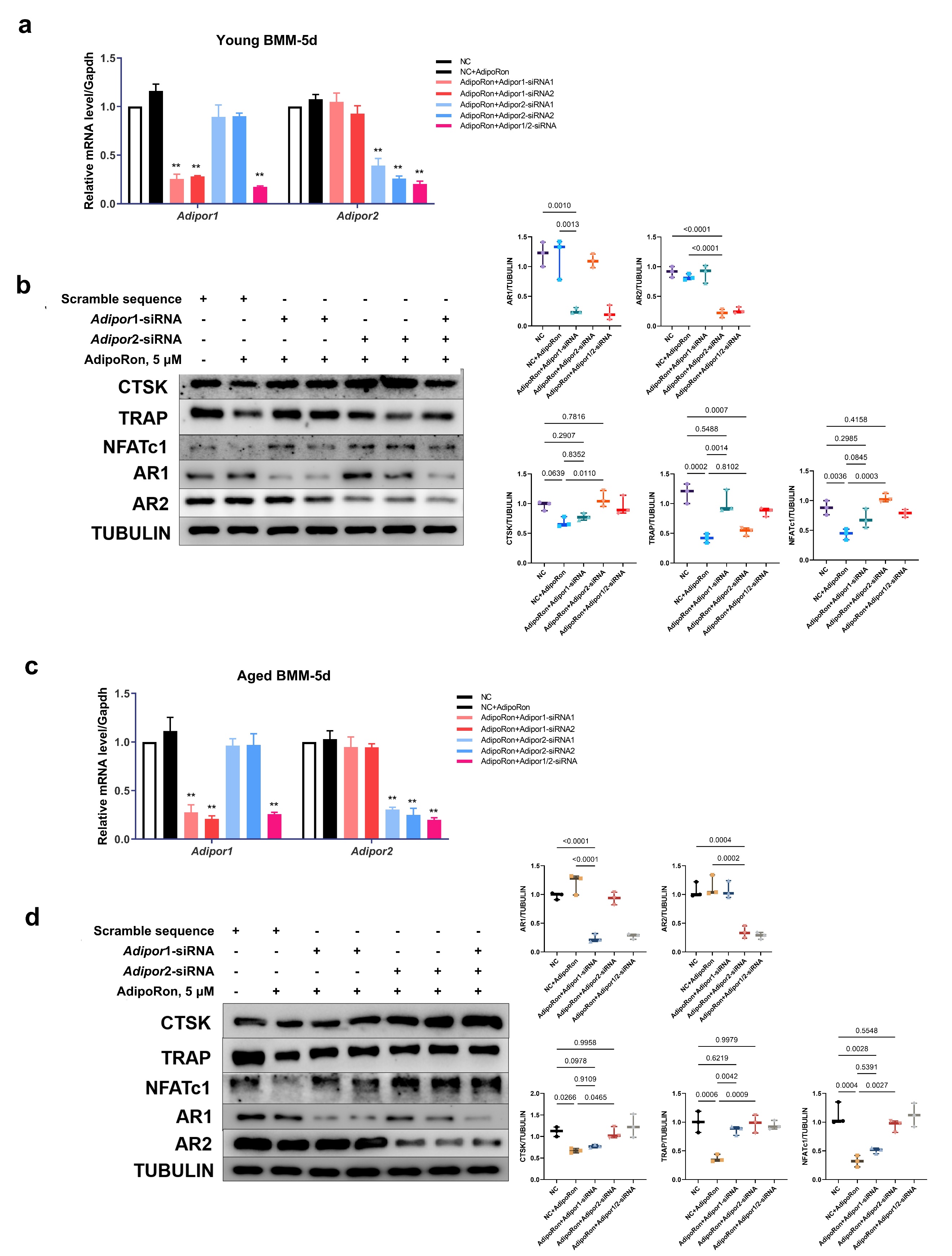


**Fig. S9.** qPCR and western blot results for young and aged BMM. **a** qPCR results of *Adipor1* and *Adipor2* for young BMM after 5 days’ OC differentiation followed by 24 hours’ siRNA treatment, n=3. Data shown as mean ± SD. *p<.05 vs. NC+AdipoRon group; **p<.01 vs. NC+AdipoRon group. **b** Immunoblot results of osteoclatogenesis related proteins for young BMM after 5 days’ OC differentiation followed by 24 hours’ siRNA treatment. n=3. **c** qPCR results of *Adipor1* and *Adipor2* for young BMM after 5 days’ OC differentiation followed by 24 hours’ siRNA treatment, n=3. Data shown as mean ± SD. *p<.05 vs. NC+AdipoRon group; **p<.01 vs. NC+AdipoRon group. **d** Immunoblot results of osteoclatogenesis related proteins for aged BMM after 5 days’ OC differentiation followed by 24 hours’ siRNA treatment. n=3.


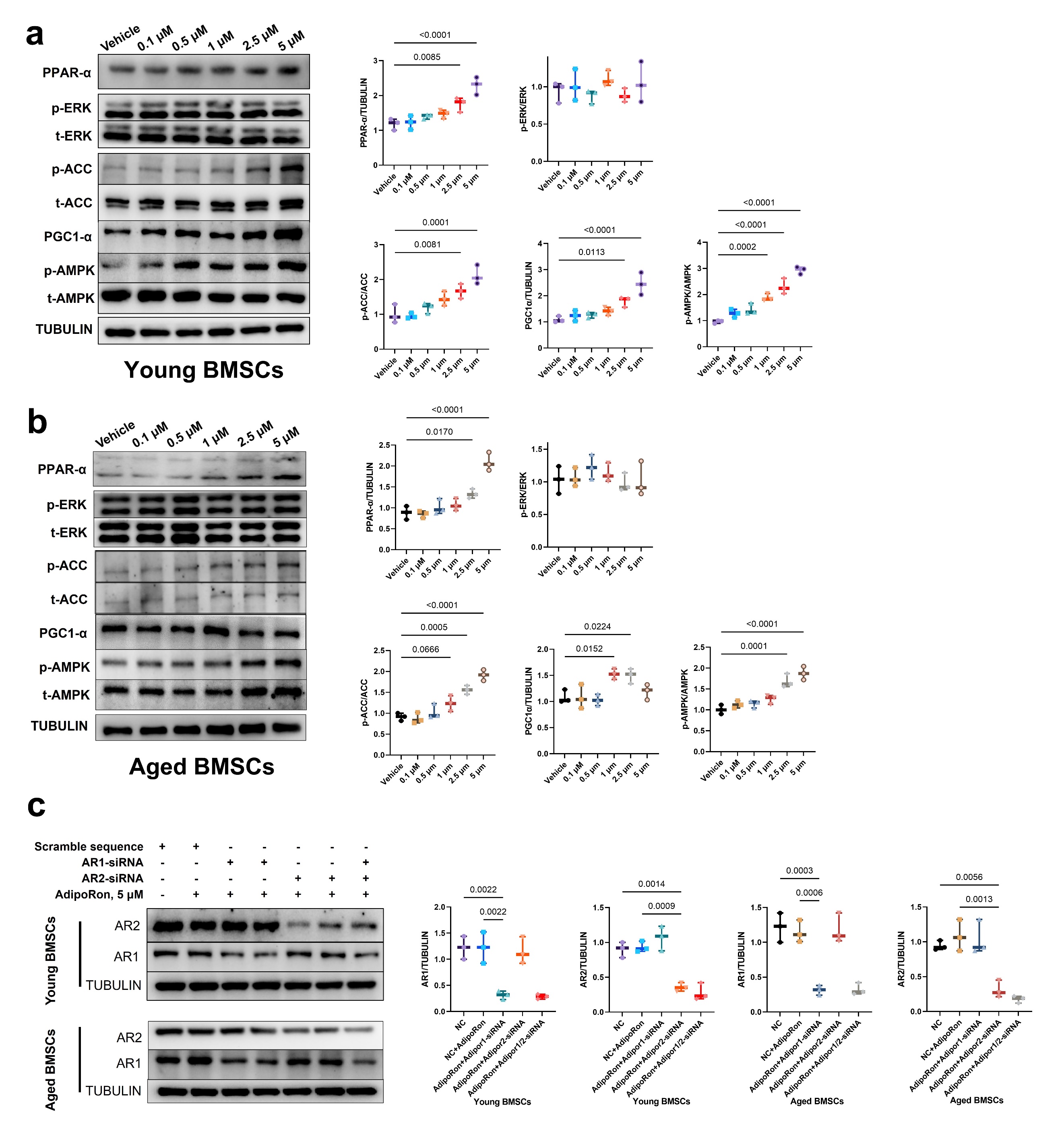


**Fig. S10.** Activated pathways in young and aged BMSCs with 24 hours’ APR treatment and siRNA efficiency confirmation. **a,b** Involved AR-Pathway in young and aged BMSCs with 24 hours’ APR treatment. **c** Expression level of AR1 and AR2 in young and aged BMSC after 24 hours’ *Adipor1* or *Adipor2* siRNA treatment followed by 24 hours’ APR treatment.


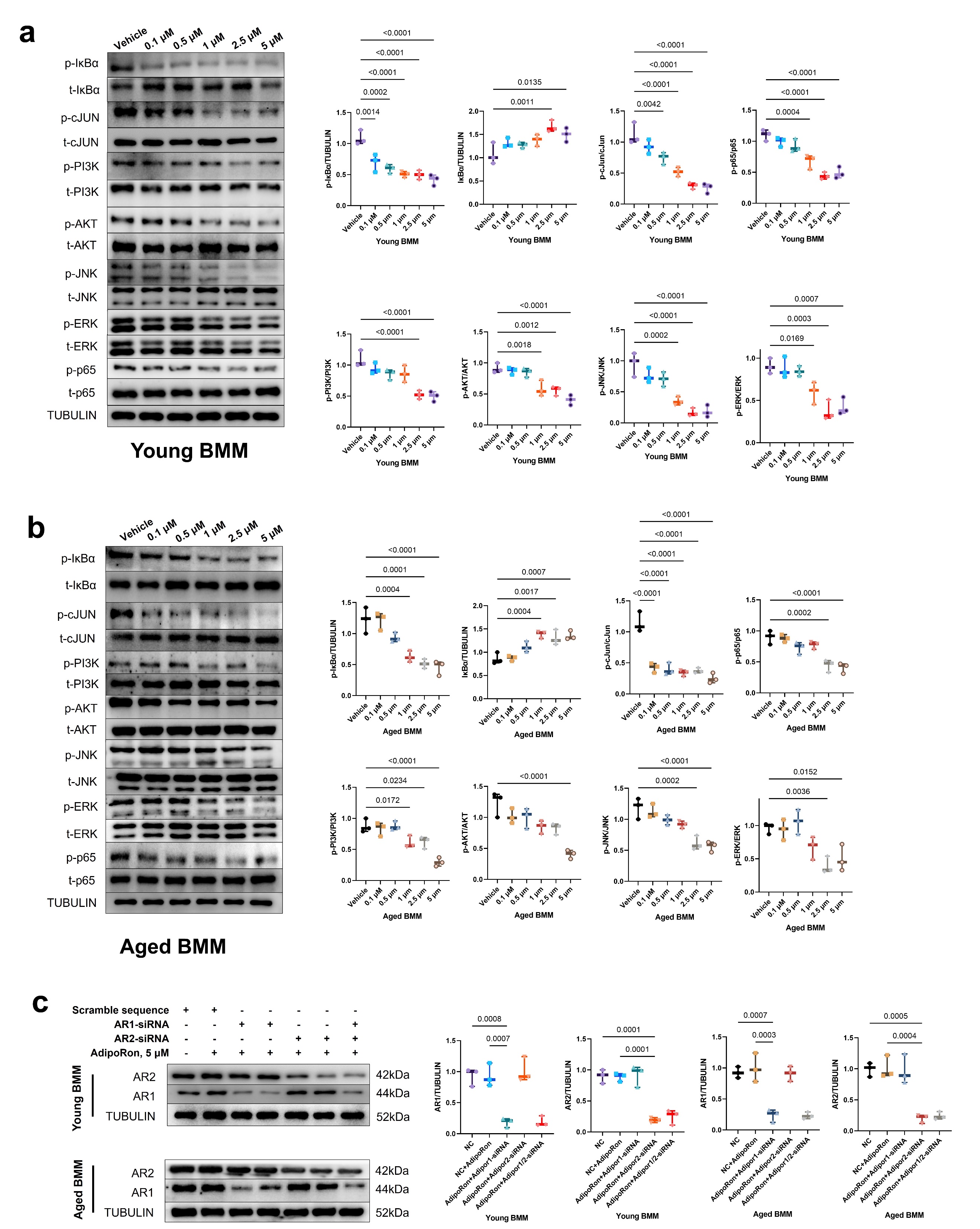


**Fig. S11.** Activated pathways in young and aged BMM with 24 hours’ APR treatment and siRNA efficiency confirmation. **a,b** Involved AR-Pathway in young and aged BMM with 24 hours’ APR treatment. **c** Expression level of AR1 and AR2 in young and aged BMM after 24 hours’ *Adipor1* or *Adipor2* siRNA treatment followed by 24 hours’ APR treatment.

| **Gene** | **Forward Primer (5’-3’)** | **Reverse Primer (5’-3’)** |
| --- | --- | --- |
| Mice | | |
| *Gapdh* | AGGTCGGTGTGAACGGATTTG | TGTAGACCATGTAGTTGAGGTCA |
| *Alp* | GACTGGTACTCGGATAACGA | TGCGGTTCCAGACATAGTGG |
| *Runx-2* | CCAACCGAGTCATTTAAGGCT | GCTCACGTCGCTCATCTTG |
| *Osx/Sp7* | ATGGCGTCCTCTCTGCTTG | TGAAAGGTCAGCGTATGGCTT |
| *Col-1* | GGTGAGCCTGGTCAAACGG | ACTGTGTCCTTTCACGCCTTT |
| *Ocn* | CAAGTCCCACACAGCAGCTT | AAAGCCGAGCTGCCAGAGTT |
| *Opn* | CAGGGAGGCAGTGACTCTTC | AGTGTGGAAAGTGTGGCGTT |
| *Opg* | CAGAGAAGCCACGCAAAAGTG | AGCTGTGTCTCCGTTTTATCCT |
| *Rankl* | CGCCAACATTTGCTTTCGG | TTTTAACGACATACACCATCAGC |
| *Trap* | CACTCCCACCCTGAGATTTGT | CATCGTCTGCACGGTTCTG |
| *Ctsk* | AATACCTCCCTCTCGATCCTACA | TGGTTCTTGACTGGAGTAACGTA |
| *Nfatc1* | GGAGAGTCCGAGAATCGAGAT | TTGCAGCTAGGAAGTACGTCT |
| *Adipor1* | GCCAAACACCGATTGGGGT | GGCTCCAAATCTCCTTGGTAGTT |
| *Adipor2* | GGAGTGTTCGTGGGCTTAGG | GCAGCTCCGGTGATATAGAGG |

**Supplementary Table 1.** Sequence of Primers

| **Antibodies** | **Company** | **Identifier** |
| --- | --- | --- |
| GAPDH | Cell signaling | 5174 |
| TUBULIN | Huabio | ER130905 |
| ALP | Abcam | ab65834 |
| RUNX-2 | Abcam | ab23981 |
| OSX | Abcam | ab209484 |
| COL-1 | Abcam | ab34710 |
| OCN | Abcam | ab93876 |
| OPN | Abcam | ab8448 |
| TRAP | Abcam | ab191406 |
| CTSK | Abcam | ab19027 |
| NFATc1 | Cell signaling | 8032 |
| AR1 | Bioss | bs-0610R |
| AR2 | Proteintech | 14361-1-AP |
| p-β/Catenin (Ser675) | Cell signaling | 4176 |
| β/Catenin | Cell signaling | 8480 |
| p-AMPK (Thr172) | Cell signaling | 50081 |
| AMPK | Cell signaling | 5831 |
| p-CaMKK2 (Ser495) | Cell signaling | 16737 |
| CaMKK2 | Cell signaling | 16810 |
| PGC-1a | Cell signaling | 2178 |
| p-ACC (Ser79) | Cell signaling | 11818 |
| ACC | Cell signaling | 3662 |
| p-mTOR (Ser2448) | Cell signaling | 2971 |
| mTOR | Cell signaling | 2972 |
| p-JNK (Thr183/Tyr185) | Cell signaling | 4668 |
| JNK | Cell signaling | 9252 |
| p-ERK (Thr202/Tyr204) | Cell signaling | 4370 |
| ERK | Cell signaling | 4695 |
| p-p38 (Thr180/Tyr182) | Cell signaling | 4511 |
| p38 | Cell signaling | 8690 |
| PPAR-a | Abcam | ab3484 |
| p-p65 (Ser536) | Cell signaling | 3033 |
| p65 | Cell signaling | 8242 |
| p-AKT (Thr308) | Cell signaling | 13038 |
| AKT | Cell signaling | 4691 |
| p-PI3K (Tyr458)/p55 (Tyr199) | Cell signaling | 17366 |
| PI3K | Cell signaling | 4292 |
| p-cJUN (Ser73) | Cell signaling | 9164 |
| cJUN | Cell signaling | 9165 |
| p-IkBa (Ser32) | Cell signaling | 5209 |
| IkBa | Cell signaling | 4812 |

**Supplementary Table 2.** Antibody list.
